# Synthetic ligands for PreQ_1_ riboswitches provide structural and mechanistic insights into targeting RNA tertiary structure

**DOI:** 10.1038/s41467-019-09493-3

**Published:** 2019-04-02

**Authors:** Colleen M. Connelly, Tomoyuki Numata, Robert E. Boer, Michelle H. Moon, Ranu S. Sinniah, Joseph J. Barchi, Adrian R. Ferré-D’Amaré, John S. Schneekloth

**Affiliations:** 1 Chemical Biology Laboratory, National Cancer Institute, Frederick, MD 21701 USA; 2 Biochemistry and Biophysics Center, National Heart, Lung and Blood Institute, Bethesda, MD 20892 USA; 3 Biomedical Research Institute, National Institute of Advanced Industrial Science and Technology (AIST), Tsukuba, Ibaraki 305-8566 Japan

## Abstract

Riboswitches are naturally occurring RNA aptamers that regulate gene expression by binding to specific small molecules. Riboswitches control the expression of essential bacterial genes and are important models for RNA-small molecule recognition. Here, we report the discovery of a class of synthetic small molecules that bind to PreQ_1_ riboswitch aptamers. These molecules bind specifically and reversibly to the aptamers with high affinity and induce a conformational change. Furthermore, the ligands modulate riboswitch activity through transcriptional termination despite no obvious chemical similarity to the cognate ligand. X-ray crystallographic studies reveal that the ligands share a binding site with the cognate ligand but make different contacts. Finally, alteration of the chemical structure of the ligand causes changes in the mode of RNA binding and affects regulatory function. Thus, target- and structure-based approaches can be used to identify and understand the mechanism of synthetic ligands that bind to and regulate complex, folded RNAs.

## Abstract (web summary)

RNA sensors—Riboswitches—respond to the binding of small molecules ligands through structure modification. Here the authors identify synthetic small molecules that bind and regulate the activity of PreQ_1_ Riboswitches despite having no obvious chemical similarity to the cognate ligand.

## Introduction

An explosion of interest in RNA biology in recent years has revealed a multitude of new regulatory functions for noncoding RNA^1,2^. Many noncoding RNAs regulate gene expression or protein function and are often dysregulated in cases of infectious disease or cancer^3,4^. The observation that regulatory, noncoding RNAs can directly influence disease states^5^ has led to the suggestion that such RNAs could be suitable as targets for small molecules^6–8^. Thus, the goal of developing RNA-binding small molecules as both therapeutics and chemical probes is an area of increasing interest. However, the discovery of molecules that bind to RNA with good affinity and specificity is a major challenge owing to RNA’s highly flexible, dynamic structure and largely solvent-exposed binding pockets, in addition to the fact that many RNAs are actively unfolded in the cell^9^. Further, understanding the function of noncoding RNA has proven highly challenging in many cases^10^. Although it is now routine to utilize structure to rationalize the function of protein-binding small molecules, high-resolution structures of RNA are often difficult to determine. The challenge in characterizing the specific contacts small molecules make with RNAs is often a substantial impediment to understanding and developing small molecule probes of RNA.

One class of regulatory, noncoding RNAs where major advances in structure determination have been seen is riboswitches. Riboswitches are structured RNA elements that occur in the untranslated regions of mRNA, most often in bacteria^11^. Riboswitches act as regulators of gene expression through recognition of a small-molecule ligand that induces a conformational change in the RNA, thereby regulating downstream gene expression^12^. Riboswitches often recognize ligands related to the function of the associated gene product^13^ and, in some examples, the ligand can be a product of the biosynthetic pathway regulated by the riboswitch. Molecules recognized can range from nucleobases, cofactors, and amino acids to metal ions^13^. These structured RNA elements have two domains, an aptamer domain that recognizes the cognate ligand through specific interactions, and an expression platform that changes conformation upon binding to modulate gene expression^12^. Riboswitch-mediated gene regulation can occur at either the transcriptional or translational level. For transcriptional riboswitches, cognate ligand binding usually promotes an RNA conformation that contains a terminator helix that triggers dissociation of RNA polymerase and leads to transcription termination upstream of the coding sequence. In the absence of the ligand, the RNA folds into an alternate antiterminator, therefore, allowing transcription of the downstream gene. In translational riboswitches, ligand binding causes a conformational change that produces a helix that occludes the Shine-Dalgarno sequence and prevents ribosomal binding. In the absence of the ligand, an alternate structure forms, increasing the availability of the ribosomal binding region and facilitating translation initiation. The ability of riboswitches to control essential bacterial genes has stimulated their evaluation as antibacterial targets. Furthermore, riboswitches also serve as important model systems for highly specific RNA-small molecule interactions^14–22^.

The PreQ_1_ riboswitch governs the expression of genes responsible for the biosynthesis of queuosine (Q)^23^. The riboswitch regulates downstream gene expression in response to its cognate ligand PreQ_1_ (7-aminomethyl-7-deazaguanine). PreQ_1_ is a modified, guanine-derived nucleobase that is incorporated into tRNAs at the wobble position, where it is further altered to produce Q^24^. The role of Q is linked to the preservation of translational fidelity and aids reading of degenerate codons. Further, removal of enzymes involved in Q biosynthesis results in multiple phenotypic defects in a variety of organisms^25,26^. This pathway is thought to be essential for bacterial virulence in some cases^27^. Many organisms contain PreQ_1_ riboswitches, and to date, three distinct PreQ_1_ riboswitch classes have been discovered that differ in both sequence and mechanism^23^. Importantly, class I PreQ_1_ (PreQ_1_-I) riboswitches, such as that found in *Bacillus subtilis* (*Bs*), are among the smallest known riboswitches, with the *Bs* riboswitch having a minimal aptamer domain of just 34 nucleotides (nt). In *Bs*, the PreQ_1_ riboswitch regulates the *queCDEF* operon that is involved in Q biosynthesis. Upon ligand binding, the *Bs*PreQ_1_ riboswitch folds into an H-type pseudoknot^28^ causing a conformational change that induces a terminator hairpin and downregulates transcription. Similarly, the PreQ_1_-I riboswitch from *Thermoanaerobacter tengcongensis* (*Tt*) also adopts an H-type pseudoknot structure^29^ in the ligand-bound state. In contrast, the *Tt* riboswitch regulates expression of a PreQ_1_ transporter^30^ and does so via translational inhibition by a conformational change that sequesters the Shine-Dalgarno sequence. The small and well-characterized aptamer domains of the PreQ_1_ riboswitches from *Bs* and *Tt* make them ideal model systems for assessing the ability to target riboswitches with synthetic small molecules to regulate downstream gene expression^31^.

In this study, we report the discovery of a new class of synthetic small molecules that bind directly to PreQ_1_ riboswitches. We performed a small molecule microarray (SMM) screen on the aptamer domain of the PreQ_1_ riboswitch found in *Bs*. Hit compounds that showed selective binding to the riboswitch over other RNAs were validated through a series of biophysical experiments. One hit compound from this screen exhibits a dissociation constant of ~ 500 nm to the *Bs*PreQ_1_ aptamer by multiple orthogonal fluorescence titrations. Further, in-line probing suggests that the synthetic ligand induces a riboswitch conformation that is different from that of the cognate ligand-bound form. The compound is also shown to bind the structurally similar, but functionally distinct *Tt*PreQ_1_ riboswitch. Co-crystal structures with this aptamer show that the compound binds in the PreQ_1_-binding site but makes key interactions with conserved nucleotides that are different from those of the cognate ligand. Importantly, in vitro transcription termination assays demonstrate that the small molecule is capable of regulating riboswitch function upon binding. Structure-guided alteration of the chemical structure of the ligand impacts both the mode of binding and activity of the ligands. The co-crystal structures will further be used for developing additional compounds that target the PreQ_1_ riboswitch and the Q biosynthetic pathway.

## Results

### SMM screening of a PreQ_1_ riboswitch aptamer

To identify drug-like small molecules that bind to the PreQ_1_ riboswitch, we used a SMM-screening strategy^32–40^. In this approach, small molecules are spatially arrayed and covalently linked to a glass surface. Next, a fluorescently tagged RNA of interest is incubated with the arrays. Slides are washed, imaged, and the fluorescence intensity is measured for each location on the array. For each compound (printed in duplicate), a composite Z-score is generated, reflecting the increase in fluorescence upon addition of labeled RNA. In parallel, other RNAs may be counter screened to evaluate selectivity and prioritize hit compounds that arise from the screen.

**Fig. 1:**
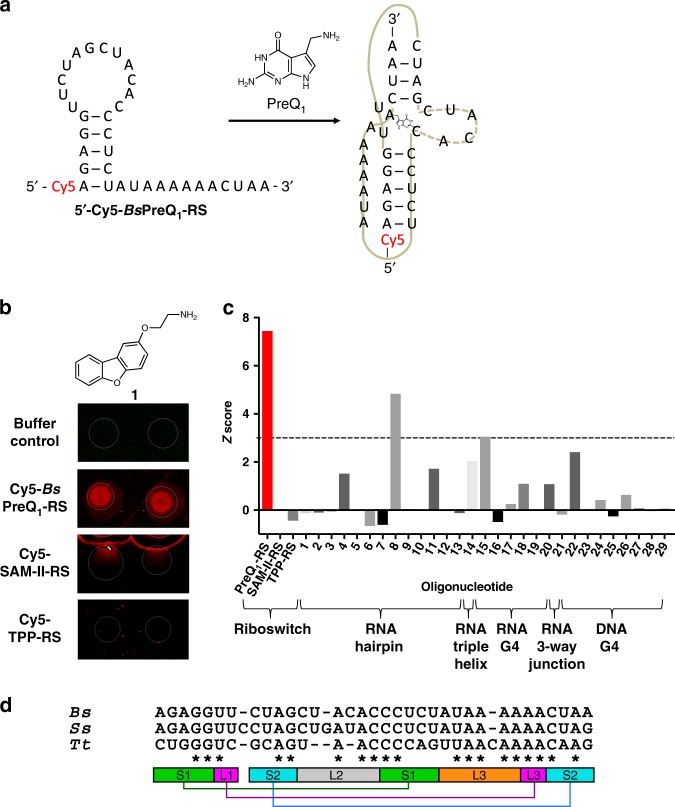
Small molecule microarray screening. **a** Sequence of the Cy5-labeled aptamer domain of the *Bs*PreQ_1_ riboswitch (5′-Cy5-*Bs*PreQ_1_-RS) used for SMM screening. The conformational change induced by PreQ_1_ binding is also shown. **b** SMM images for the hit compound **1** (compound is printed in duplicate). Spot diameter (white circle) is 150 µm. **c** Selectivity of the hit compound across various oligonucleotides screened using the SMM platform (Supplementary Table 2) as measured by composite Z-score. Horizontal line corresponds to a “hit” cutoff of 3. **d** Sequence alignment of *Bs*, *Ss*, and *Tt*PreQ_1_ riboswitch aptamers. Conserved nucleotides are marked by asterisks. Secondary structure of the class I PreQ_1_ riboswitch aptamers is below the alignment and base-pairing interactions are indicated by colored bars

Toward this end, we designed a 5′-Cy5-labeled RNA consisting of the 34-nt aptamer domain of the PreQ_1_ riboswitch from *Bs* (5ʹ-Cy5-*Bs*PreQ_1_-RS, Fig. 1A)^30^. Once purity was confirmed by gel electrophoresis, the RNA was annealed and incubated on SMM slides to perform the screen. In parallel, we also screened analogous 5′-Cy5-labeled constructs consisting of aptamer domains from *S*-adenosylmethionine (SAM-II)^41^ and thiamine pyrophosphate (TPP)^42^ riboswitches (Supplementary Table 1). Each of these three aptamer domains are known to recognize different biologically active ligands and have well defined, complex three-dimensional structures, making them suitable controls for selectivity. To further measure selectivity, the Z-score for each compound is compared across many different SMM screens (in this case 29 different oligonucleotides, Supplementary Table 2). We identified 243 hits from a collection of 26,227 compounds screened in the SMM, for an initial hit rate of 0.93%. After ruling out compounds that bound promiscuously to other riboswitches or other RNAs and DNAs previously screened against the library, we generated a list of 86 candidate compounds for further study (Supplementary Table 3). The TPP and SAM-II riboswitches had slightly fewer numbers of “selective” hits, with rates of 51 hits (0.19%) and 61 hits (0.23%), respectively. Twenty hits identified as selective for the PreQ_1_ aptamer were purchased for further analysis (Supplementary Table 4 and Supplementary Fig. 1). As a representative example, direct binding on SMM slides (Fig. 1B) and selectivity data (Fig. 1C) are shown for compound **1**.

### Ligand observed NMR validates binding

**Fig. 2:**
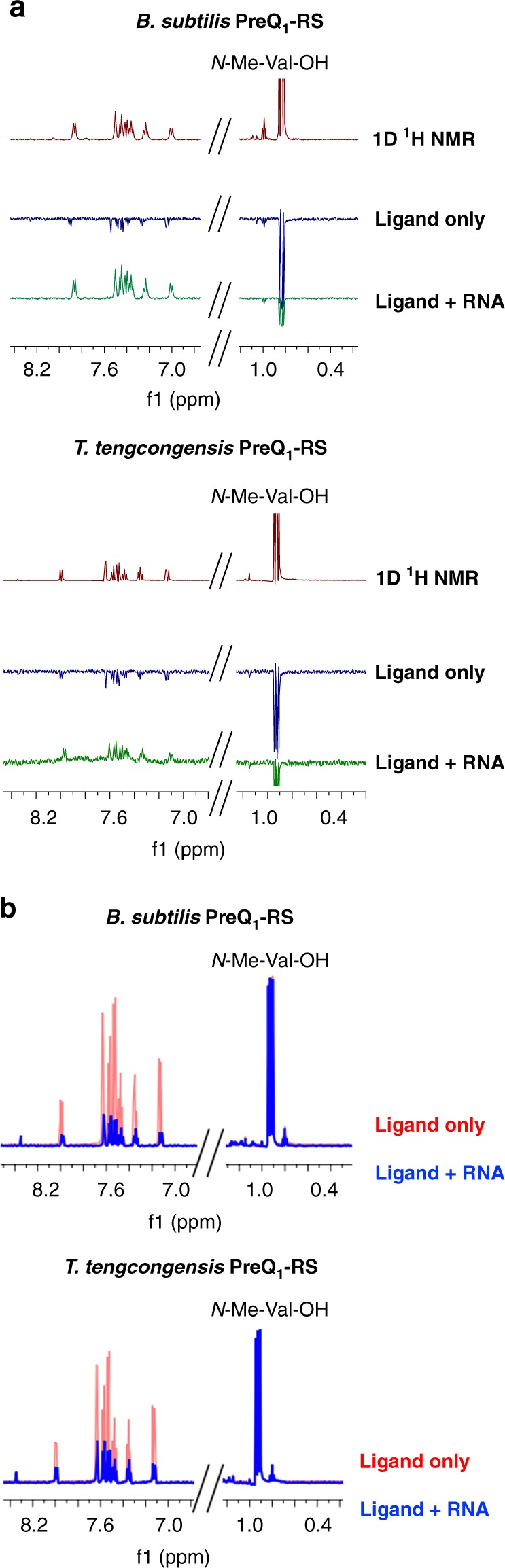
NMR validation of hit compound **1**. **a** ^1^H NMR of **1** and *N*-methyl-l-valine (non-binding control) (Top, red spectrum), WaterLOGSY NMR of **1** and *N*-methyl-l-valine in the absence (middle, blue spectrum) and presence (bottom, green spectrum) of either the *Bs* or *Tt*PreQ_1_ riboswitch aptamer. **b** CPMG of **1** and *N*-methyl-l-valine in the absence (red) and presence (blue) of either the *Bs* or *Tt*PreQ_1_ riboswitch aptamer

Each of the purchased compounds was evaluated using Water-Ligand Observed via Gradient Spectroscopy (WaterLOGSY) NMR^43^. In this experiment, each compound was subjected to a standard ^1^H NMR pulse sequence (to assess solubility in aqueous buffer), WaterLOGSY without RNA (to assess aggregation), and WaterLOGSY in the presence of unlabeled *Bs*PreQ_1_-RS RNA (to assess binding to the riboswitch aptamer) (Fig. 2A). For compounds that bind directly to the RNA, peaks phase positively only in the presence of RNA. In contrast, aggregating compounds phase positively even in the absence of RNA. Peaks for compounds that are soluble but do not bind to the RNA are phased negatively in both cases (as can be seen with *N*-methyl-l-valine, included here as a convenient internal, non-binding control). From these experiments, we identified five compounds with suitable solubility and RNA binding in the solution phase. We also evaluated binding of each compound against 31 different structured RNAs and DNAs from previous SMM screens, where compound **1** was found to have the best profile of selective binding (Fig. 1C).

To further validate these results, we also evaluated **1** by WaterLOGSY NMR with a second PreQ_1_ riboswitch aptamer domain from *Tt* (unlabeled *Tt*PreQ_1_-RS, Supplementary Table 1)^29^. Although divergent in sequence and genetic control mechanism^44^, this riboswitch also recognizes the same cognate ligand and has a mostly conserved binding site. Gratifyingly, **1** also bound directly to the *Tt*PreQ_1_ aptamer domain by WaterLOGSY (Fig. 2A). In addition to WaterLOGSY experiments, we evaluated the behavior of **1** in Carr-Purcell-Meiboom-Gill (CPMG) ^1^H NMR experiments, which can be used to observe alterations in T_2_ relaxation times of small molecules that bind large molecules in solution (with binding shortening relaxation time)^45,46^. Here, **1** was subjected to a CPMG pulse sequence in the absence and presence of both the *Bs* and *Tt* aptamers used in the WaterLOGSY experiments. In both cases, substantial peak attenuation of **1** with no attenuation of the control was observed in the presence of RNA, indicating shortening of the T_2_ relaxation time of protons in **1** (Fig. 2B). Thus, CPMG experiments further validate the binding of **1** to both aptamers. Because the two aptamers have conserved binding sites but diverge moderately in structure, this suggested that **1** could potentially share a binding site with the cognate ligand.

### Fluorescence titrations demonstrate submicromolar binding

**Fig. 3:**
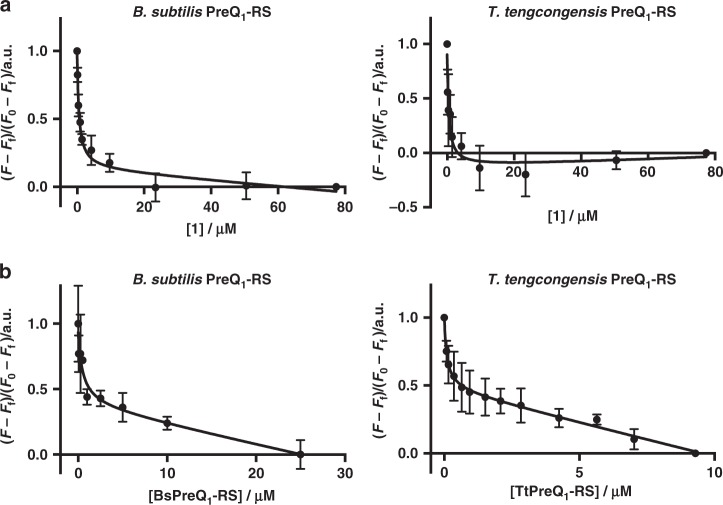
Affinity of **1** for PreQ_1_-RS aptamers. **a** Fluorescence intensity assay of 5′-Cy5-labeled *Bs*PreQ_1_-RS or 5′-AlexaFluor 647-labeled *Tt*PreQ_1_-RS RNA in the presence of increasing concentration of **1**. **b** Inherent fluorescence titration of **1** with increasing concentration of unlabeled *Bs*PreQ_1_-RS or *Tt*PreQ_1_-RS RNA. Error bars indicate the standard deviation determined from three independent measurements. Source data are provided as a Source Data file

To estimate the affinity of **1** to the PreQ_1_ riboswitch aptamer domain, we used orthogonal fluorescence titrations. First, we measured changes in fluorescence of the Cy5-labeled *Bs* aptamer domain construct used for SMM screening (5ʹ-Cy5-*Bs*PreQ_1_-RS) as a function of compound concentration. In parallel, we evaluated effects on an AlexaFluor 647-labeled *Tt* aptamer domain construct (5ʹ-AF647-*Tt*PreQ_1_-RS, Supplementary Table 1) to both measure affinity and further rule out effects associated with the fluorophore (Fig. 3A). In the case of the *Bs* aptamer, we measured an apparent dissociation constant (*K*_D_) of 534 ± 123 nm for **1**. Similarly, **1** had a *K*_D_ of 457 ± 202 nm for the *Tt* aptamer domain. For comparison, we also measured the affinity of PreQ_1_ itself in this assay. The affinity of PreQ_1_ for the *Bs* aptamer was 4.1 ± 0.6 nM, and for the *Tt* aptamer it was 2.8 ± 0.4 nm (Supplementary Fig. 2). This is in good agreement with literature values measured by other methods^29,30,44^. Owing to the presence of a conjugated π-electron system, we evaluated **1** for inherent fluorescence and found that **1** was fluorescent, with *λ*_ex_ = 300 nm and *λ*_em_ = 340 nm. Thus, by holding the concentration of **1** constant and titrating in increasing quantities of unlabeled riboswitch aptamers, we could measure *K*_D_ values by observing changes in fluorescence as a function of RNA concentration (Fig. 3B). Here, we measured a *K*_D_ of 490 ± 368 nm for the *Bs* aptamer domain and 99 ± 38 nm for the *Tt* aptamer domain, respectively. Thus, there is good agreement for *K*_D_ measurements between multiple different techniques. In addition to the selectivity measured by comparing different SMM screens (Fig. 1C), we also used fluorescence titration to evaluate the binding of **1** to tRNA. Upon titrating tRNA into a solution of **1**, only non-specific binding was observed, and fluorescence quenching did not fit to a 1:1 binding model, indicating only weak binding to tRNA at high micromolar levels (Supplementary Fig. 3).

### Ligand induces conformational change to riboswitch aptamers

**Fig. 4:**
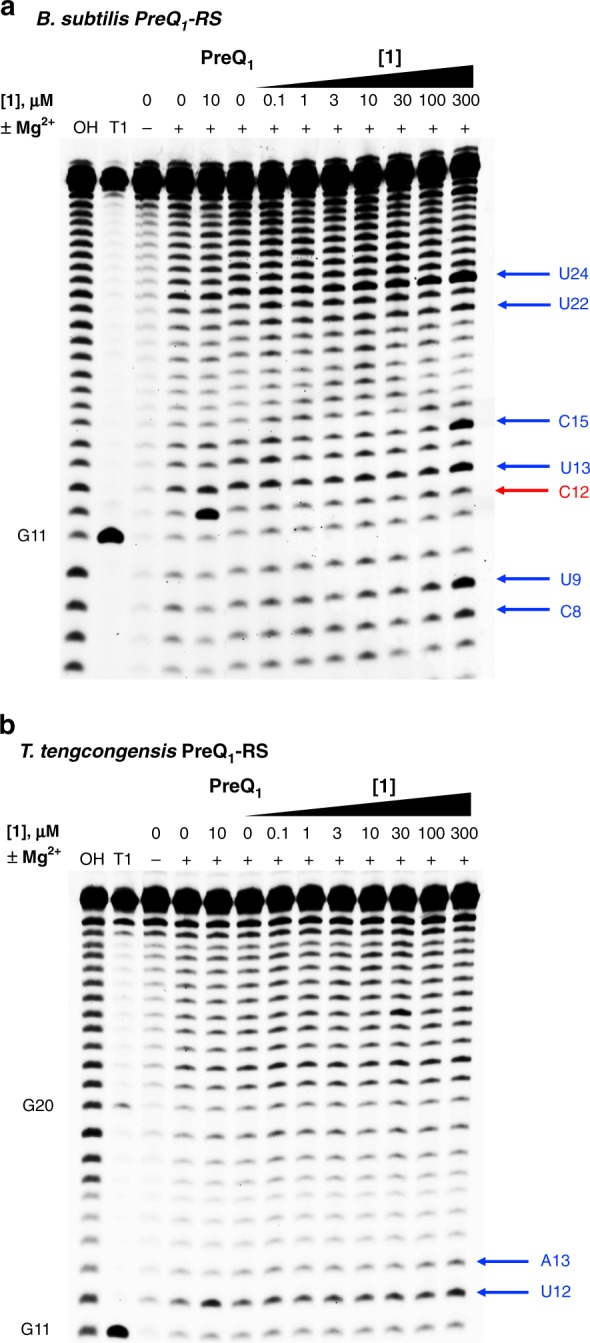
Ligand-induced conformational changes. In-line probing of **a** 5′-AlexaFluor 647-labeled *Bs*PreQ_1_-RS RNA and **b** 5′-AlexaFluor 647-labeled *Tt*PreQ_1_-RS RNA after treatment with **1** at increasing concentrations or a DMSO control in the absence (–) or presence (+) of 1 mm MgCl_2_. Treatment with PreQ_1_ at a concentration of 10 μm is used as a positive control. OH and T1 are a partial alkaline hydrolysis ladder and ribonuclease T1 digestion, respectively. Arrows designate nucleotide positions where the cleavage efficiency was significantly altered by compound treatment (blue) or preQ_1_ treatment (red)

A key aspect of riboswitch biochemistry is that biological effects are driven by ligand-induced conformational changes in the RNA upon binding. Thus, we aimed to evaluate the effects of **1** on the conformation of the PreQ_1_ aptamers by in-line probing experiments^30^. In-line probing experiments are routinely used to monitor alterations of riboswitch conformation and can report on ligand-induced effects on RNA structure. First, we performed in-line probing using a fluorescently tagged 36-mer *Bs* aptamer domain (5ʹ-AF647-*Bs*PreQ_1_-RS, Supplementary Table 1) with PreQ_1_. Consistent with previous reports using a radiolabeled 36-mer construct^30^, we observed an increase in cleavage at C12 and a corresponding decrease in cleavage at U32 (based on 34-mer numbering, Fig. 1D). Thus, in-line probing using a fluorescently tagged construct accurately matches literature reports on ligand-induced conformational changes. Next, we performed an analogous experiment in the presence of synthetic ligand **1** (Fig. 4A). In contrast to the cognate ligand, **1** caused an increase in cleavage at C8, U9, U13, C15, U22, and U24, whereas it did not induce prominent increase in cleavage at C12. Although these changes were consistent from experiment to experiment, smaller changes in cleavage at other residues (for example nucleotides 17–19) were less consistent from experiment to experiment and could not be attributed to a specific binding interaction. Thus, although the compound binds directly to the *Bs* aptamer domain and alters the conformation of the RNA, the effects of **1** in this assay are distinct from those of PreQ_1_. We also performed in-line probing with the *Tt* riboswitch (5ʹ-AF647-*Tt*PreQ_1_-RS, Supplementary Table 1). In this case, with PreQ_1_ the most notable increase in cleavage occurred at U12 (Fig. 4B). Upon addition of compound **1**, a similarly small increase in cleavage occurred at U12 and A13. Thus, **1** may induce a conformational change in the *Tt* aptamer similar to PreQ_1_. The *Bs* and *Tt* riboswitches have been previously described to have similar conformational ensembles but differ in their ability to recognize ligands by conformational selection and induced fit mechanisms, respectively^44^. This distinction may be reflected in the effects observed in in-line probing experiments.

### Ligand modulates riboswitch transcriptional termination

**Fig. 5:**
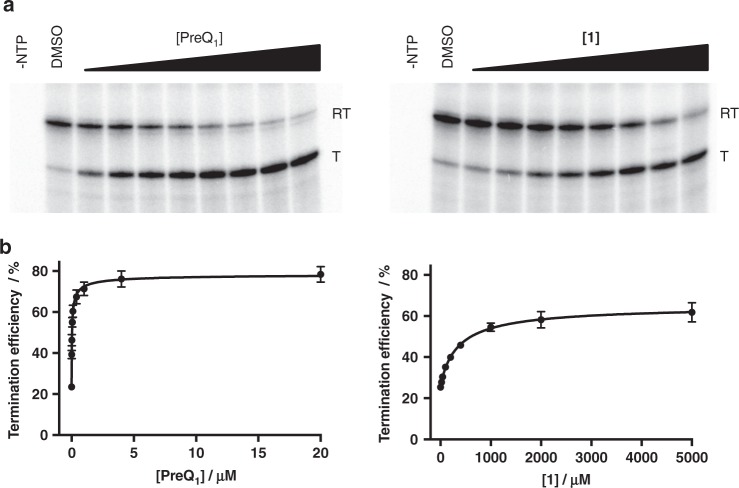
Transcription termination assay of **1**. **a** Representative gel images of the ^32^P-labeled RNA products of in vitro transcription of the *Ss* PreQ_1_-RS template in the presence of increasing concentrations of PreQ_1_ (left) and **1** (right). Bands corresponding to the read-through transcription product (RT) and terminated transcription product (T) are indicated. Full gel images are provided in the Supplementary Information. **b** Quantification of transcription termination efficiency of increasing concentrations of PreQ_1_ (left) or **1** (right). Error bars indicate standard deviation, *n* = 3. Source data are provided as a Source Data file

Having demonstrated that **1** binds and causes conformational changes in both the *Bs* and *Tt* aptamers, we next evaluated **1** in a functional assay. As described above, the *Bs* riboswitch regulates the transcription of downstream genes upon ligand binding. Thus, we examined the capacity of **1** to modulate transcriptional termination. DNA templates were designed containing the aptamer domain and terminator hairpin of the BsPreQ_1_ riboswitch, followed by an elongation sequence (BsPreQ_1_ TTA, Supplementary Table 5). In vitro transcription of these templates produces an RNA sequence containing the riboswitch. This transcription can either be halted at the terminator hairpin in the presence of the cognate ligand or produce a read-through product that is transcribed to the end of the template. Despite repeated attempts, efforts to observe transcriptional termination of the *Bs* riboswitch by PreQ_1_ were unsuccessful in this assay. *Staphylococcus saprophyticus* (*Ss*) also contains a related PreQ_1_ riboswitch sequence with a conserved binding site that functions by transcriptional termination (Fig. 1D). Dose-dependent transcriptional termination was observed with a template containing the *Ss* riboswitch (*Ss* PreQ_1_ TTA, Supplementary Table 5). Near-complete termination was observed at the highest PreQ_1_ concentrations tested with an EC_50_ for PreQ_1_ of 36 ± 5 nm (Fig. 5). Similarly, addition of increasing concentrations of **1** also resulted in near-complete termination of transcription with an EC_50_ of 359 ± 23 μμ. Thus, **1** functions by a mechanism similar to PreQ_1_ itself. We also confirmed that **1** binds to the *Ss* aptamer by WaterLOGSY experiments (Supplementary Fig. 4). In addition, the *K*_D_ of **1** for the *Ss* aptamer was determined to be by 42 ± 6 nm by fluorescence titration (Supplementary Fig. 5). As a further control, the chemically unrelated ligand 5-aminoimidazole-4-carboxamide ribonucleotide, which binds to other riboswitches^47^, was tested in transcriptional assays and had no effect on the *Ss* reporter construct (Supplementary Fig. 7).

### X-Ray crystal structure of the ligand/aptamer complex

To establish a molecular basis for its selective binding, we solved crystal structures of **1** bound to the *Tt*PreQ_1_ riboswitch aptamer domain. Initial efforts using the wild-type *Tt*PreQ_1_ riboswitch aptamer domain failed, yielding structures indistinguishable from the previously reported ligand-free aptamer domain structure of the RNA, in which the nucleobase of A14 occupies the PreQ_1_-binding site^29^. To alleviate competition by the intramolecular interaction with binding of the exogenous ligand, we designed aptamer domains in which the nucleobase at position 14, as well as one or two adjacent disordered loop nucleobases were removed. (ab13_14 and ab13_14_15, Supplementary Table 1). Co-crystal structures of these abasic *Tt*PreQ_1_ riboswitch aptamer domains complexed with **1** (ab13_14-**1**, and ab13_14_15-**1**) were determined by the molecular replacement (MR) method and both refined at 1.8 Å resolution. These two structures are near-identical (r.m.s.d. is 0.14 Å for 638 non-hydrogen atom pairs). The following discussion focuses on the ab13_14-**1** complex. We also determined the crystal structure of ab13_14 in complex with PreQ_1_ (ab13_14-PreQ_1_) at 1.69 Å resolution, for comparison (Methods and Supplementary Table 7).

**Fig. 6:**
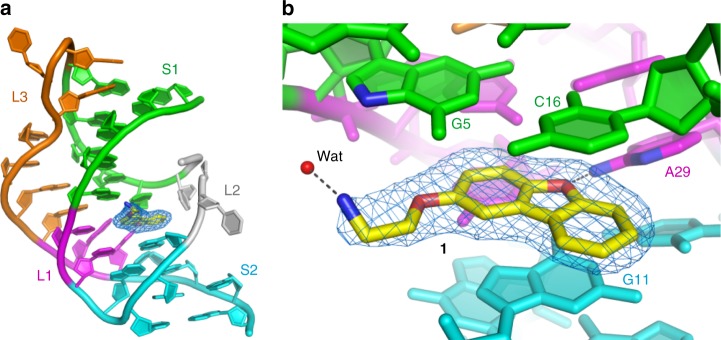
Co-crystal structure of the aptamer complexed with **1**. **a** Overall structure of the complex. S1, S2, L1, L2, and L3 are colored green, cyan, magenta, gray, and orange, respectively. The nucleotides between L3 and S2, which interact with L1, are also in magenta. Unbiased |*F*_o_|−|*F*_c_| electron density map for the compound **1** (blue mesh) contoured at 3.0 σ. **b** Detail of the ligand-binding site. Nucleotides that interact with **1** are labeled. Dotted lines denote hydrogen bonds between **1** and a bound water molecule and with A29

Unbiased |*F*_o_|−|*F*_c_| residual electron-density maps unambiguously located **1** at the interhelical interface of stems S1 and S2, surrounded by the L2 and L3 loops, in both co-crystal structures (Fig. 6A, ab13_14-**1**; Supplementary Fig. 8, ab13_14_15-**1**). Thus, the synthetic ligand occupies a position similar to that of the cognate ligand PreQ_1_. The dibenzofuran of **1** is sandwiched between G11 and the G5•C16 pair, with the furan oxygen engaging in a hydrogen bond (3.2 Å) with the N6 atom of A29 (Fig. 6B and Supplementary Fig. 5). The same oxygen is in van der Waals contact with the N1 atom of A29 (3.7 Å) The nucleotides that interact with **1** are phylogenetically conserved among PreQ_1_ riboswitches (Fig. 1D), being responsible for PreQ_1_ recognition (Supplementary Fig. 9)^28^. In contrast to the solvent-inaccessible heterocycle of **1**, its amine-bearing sidechain (largely coplanar with the dibenzofuran heterocycle) is solvent exposed, with the amine hydrogen bonding to water, and therefore not directly recognized by the RNA (Fig. 6B). However, to avoid steric clash with the L2 loop, **1** must bind with its sidechain emerging from the PreQ_1_-binding pocket away from L2 loop of the riboswitch. Of note, lack of direct recognition of the amine of **1** provides a plausible explanation for how the binding event occurred when **1** was chemically conjugated to the SMM slide. Although the sugar of residue 13 of the ab13_14-**1** structure lacked electron density and is presumed disordered, in the ab13_14_15-**1** structure, the ribose ring of residue 13 is ordered and in van der Waals contact with ring C of the dibenzofuran of **1** (Supplementary Figure 8).

### Structure-guided changes alter mode of binding and activity

**Fig. 7:**
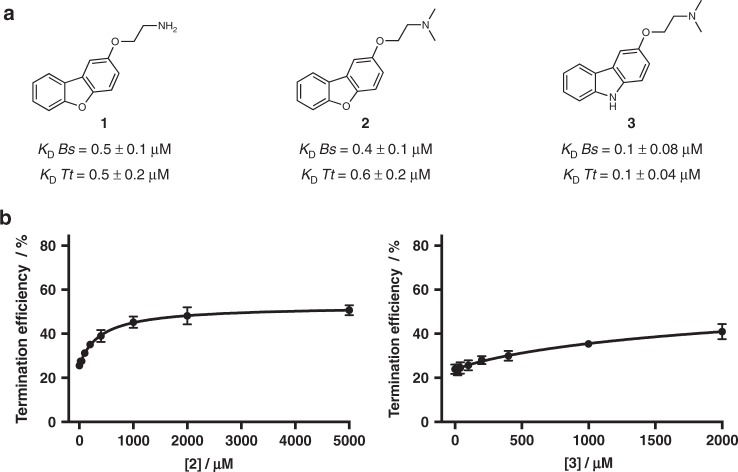
Evaluation of modified analogs of **2** and **3**. **a** Apparent *K*_D_ values for **1**–**3** as determined by fluorescence intensity assays with 5′-Cy5-*Bs*PreQ_1_-RS and 5′-AlexaFluor 647-*Tt*PreQ_1_-RS RNA. Values are the mean ± s.d. (error bars) of three replicate measurements. **b** Quantification of termination efficiency of increasing concentrations of **2** (left) and **3** (right) in transcription termination assays of a DNA template containing the *Ss* PreQ_1_ riboswitch. Error bars indicate the standard deviation determined from three independent measurements. Source data are provided as a Source Data file. Gel images are provided in the Supplementary Information

Next, we asked whether alteration of the chemical structure of **1** would have effects on riboswitch recognition or activity. We hypothesized that alteration of the basicity of the pendant amine of **1** might enable it to engage in hydrogen-bonding contacts with the RNA. Thus, we prepared the dimethylamino derivative **2**, containing a more basic tertiary amine (Fig. 7A). We evaluated the affinity of **2** for both the *Bs* and *Tt* aptamers by monitoring changes in fluorescence of the 5ʹ-Cy5-*Bs*PreQ_1_-RS and 5ʹ-AF647-*Tt*PreQ_1_-RS RNAs (Supplementary Table 1) upon titration with **2** and determined affinities of 0.4 ± 0.1 μm and 0.6 ± 0.2 μm, respectively (Supplementary Fig. 10). In addition to the pendant amine, we asked whether alteration of the core heterocyclic scaffold would have effects on RNA binding. We noted that in the structure of **1** bound to the riboswitch, the exocyclic amine of A29 makes a hydrogen bond with the oxygen atom of the dibenzofuran. In this structure, A29 is not coplanar with **1** and sits at an angle of 40°, where N1 and N6 bisect the plane of the dibenzofuran core. Nucleotides are amphiphilic and are capable of serving as both hydrogen bond donors and acceptors. We therefore synthesized a carbazole derivative of **2**, compound **3** (Fig. 7A), replacing the dibenzofuran oxygen with a nitrogen atom (that may potentially donate a hydrogen bond). Affinities of **3** for the *Bs* and *Tt* riboswitch aptamer domains were measured to be 0.1 ± 0.08 μm and 0.1 ± 0.04 μm, respectively, using fluorescence titrations (Supplementary Fig. 10). Thus, all three compounds have similar affinities for the riboswitch aptamers. Finally, **2** and **3** were evaluated in transcriptional termination assays (Fig. 7B, Supplementary Fig. 11). Here, **2** behaved similarly to **1** in terms of maximal effect. However, **3** was markedly inferior in the functional assay.

**Fig. 8:**
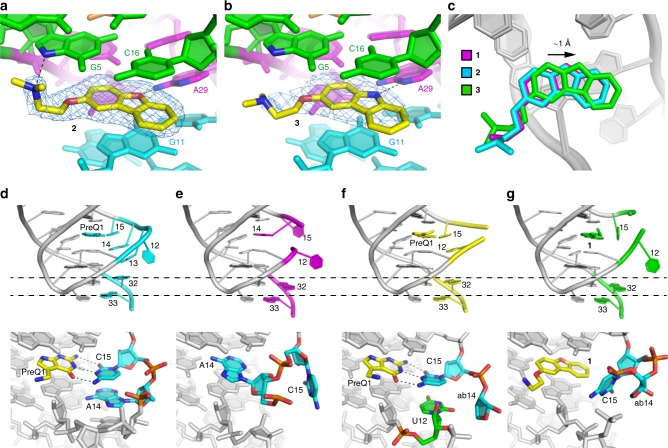
Structural analysis of the bound *Tt*PreQ_1_ aptamers. **a** Ligand-binding site of the ab13_14-**2** co-crystal structure, superimposed on the |*F*_o_|−|*F*_c_| electron-density map calculated before addition of the ligand to the crystallographic model (blue mesh, 3.0 σ contour). Hydrogen bonds are indicated as dotted lines. **b** Ligand-binding site of the ab13_14_15-**3** co-crystal structure. The |*F*_o_|−|*F*_c_| electron density map for the compound is colored blue and contoured at 3.0 σ. **c** Comparison of binding modes of **1** (magenta), **2** (cyan), and **3** (green). Ligand-binding site and continuous base stack of the aptamer domains of the **d** WT-PreQ_1_, **e** WT free, **f** ab13_14-PreQ_1_, and **g** ab13_14-**1** forms. (Upper) Cartoon representations with ligands and key nucleotides labeled and colored cyan, magenta, yellow, and green in the WT-PreQ_1_, WT free, ab13_14-PreQ_1_, and ab13_14-**1** forms, respectively. To compare the locations of A32 and G33 among these structures, dashed lines are indicated. (Lower) Detail of the ligand-binding site. PreQ_1_ and **1** are in yellow. Key nucleotides responsible for forming the base stack are colored cyan. U12 in the ab13_14-PreQ_1_ structure, which occupies a similar location to A14 in the WT-PreQ_1_ structure, is in green. Hydrogen bonds between PreQ_1_ and C15 are indicated as dotted lines

To further understand the interactions of **2** and **3** with the riboswitches, we also solved crystal structures of each ligand in complex with the *Tt* riboswitch. The co-crystal structures of abasic mutant riboswitch aptamer domains bound to **2** and **3** (ab13_14-**2** and ab13_14_15-**3**, respectively) were solved by the MR method and refined at 1.94 Å and 2.56 Å resolution, respectively (Methods and Supplementary Table 7). The overall structure of ab13_14-**2** is similar to that of the riboswitch bound to **1**, with **2** in a similar binding pose, directly in the PreQ_1_ binding site (Fig. 8A). In this structure, the sidechain of **2** bends upwards at the methylene preceding the amine, bringing the pyramidal tertiary amine of **2** within hydrogen-bonding distance (2.9 Å) of the N7 atom of the phylogenetically conserved G5 of the riboswitch (Fig. 1D). In contrast, the primary amine of **1** lies 3.6 Å from the same RNA atom. Next, we solved the crystal structure of **3** in complex with the *Tt* riboswitch aptamer domain. Similar to **1** and **2**, compound **3** occupies the PreQ_1_-binding site (Fig. 8B). However, in this structure, **3** has shifted by ~1 Å in the direction of RNA loop L2 (Fig. 8C) to support a hydrogen bond between the new donor atom in the heterocycle and the RNA (the distances between the carbazole nitrogen atom of **3** and the N1 and N6 atoms of A29 are 3.2 Å and 3.5 Å, respectively). This confirms that alteration of the chemical structure of the initial hit can lead to changes in binding modes and somewhat altered affinity. Possibly owing to the ~1 Å shift in its binding pose (Fig. 8C), the conformation of the sidechain of the bound **3** resembles that of the bound **1** rather than that of **2**. The sidechain of the bound **3** is largely coplanar with the carbazole, exposed to solvent, and does not lie within hydrogen-bonding distance of G5 of the RNA.

## Discussion

In this work, we report the discovery of synthetic ligands that bind to different PreQ_1_ riboswitch aptamer domains. Compounds discussed here are characterized by multiple orthogonal biophysical techniques, and exhibit submicromolar dissociation constants to the RNA. Furthermore, structural probing experiments indicate clear ligand-induced conformational changes. As a consequence of binding, the compounds exhibit activity in transcriptional termination assays, inducing premature termination analogous to those produced by the cognate ligand itself. Thus, the compounds function by a mechanism similar to PreQ_1_, despite having no obvious chemical similarity. Structural characterization of the ligand-aptamer complexes by X-ray crystallography provides insights into both the mode of recognition and mechanism of action of these compounds.

Structural comparison between ab13_14-**1** and the PreQ_1_-bound wild-type *Tt*PreQ_1_ riboswitch (referred to as WT-PreQ_1_) reveals conformational differences in four nucleotides (positions 12–15) of the L2 loop and two nucleotides at the 3’-end (A32 and G33) of these complexes. In the WT-PreQ_1_ structure, C15 recognizes PreQ_1_ through Watson-Crick base pairing and serves as a starting point of the consecutive base stack, C15-A14-A13-A32-G33. The last two nucleotides (A32 and G33) of the base stack constitute the Shine-Dalgarno sequence and form stem S2 by base-pairing with A10 and C9, respectively. Therefore, the continuous base stack is proposed to be crucial to inhibit gene expression through sequestrating the Shine-Dalgarno sequence from the recognition by the ribosome. Because **1** is bulkier than PreQ_1_, compound-binding interferes with the correct positioning of C15. Consequently, C15 in the ab13_14-**1** structure shifts position and its base is placed perpendicular to that of the WT-PreQ_1_ structure to escape the steric clash with **1** (Fig. 8D and G). Moreover, A32 and G33 are located downward (far away from ligand binding site) compared to those of the WT-PreQ_1_ structure. Therefore, the ab13_14-**1** structure is similar to the free state of the riboswitch, despite the difference that the ligand-binding site was occupied by A14 in the latter structure (Fig. 8E and G). The striking contrast between the ab13_14-**1** and WT-PreQ_1_ structures is predominantly attributable to the binding of the bulkier compound to the riboswitch. Consistent with this, the crystal structure of ab13_14-PreQ_1_ showed a similar conformation to WT-PreQ_1_ rather than ab13_14-**1**, where C15 in ab13_14-PreQ_1_ is well superimposed to that in WT-PreQ_1_ and A32 and G33 in ab13_14-PreQ_1_ shift toward the ligand-binding site compared with the ab13_14-**1** structure (Fig. 8D–G and Supplementary Fig. 5). In addition, U12 of ab13_14-PreQ_1_ is placed in a similar location to A14 of WT-PreQ_1_ and hydrogen bonds with G11 in order to compensate for the absence of the base at position 14. Therefore, PreQ_1_ binding to the ligand binding site results in a conformation similar to WT-PreQ_1_ despite the absence of the bases at positions 13 and 14. From these findings, **1** binding to the wild-type *Tt*PreQ_1_ riboswitch may induce a suboptimal formation of the continuous base stack that transmits the ligand binding information to the expression platform of the riboswitch. This suboptimal conformation may lead to the decreased maximum termination efficiency in the transcription termination assay for **1** in comparison with the cognate ligand.

Like the translational class I PreQ_1_ riboswitch, transcriptional class I PreQ_1_ riboswitches also have a continuous base stack from the ligand-binding site to the expression platform. Given that the transcriptional and translational PreQ_1_ riboswitches control ON/OFF switching using similar structural motifs, the **1**-bound *Tt*PreQ_1_ riboswitch structure can explain why this compound has an EC_50_ of 359 ± 23 μm in transcription termination assays (*Ss* aptamer). This value is considerably higher than that for PreQ_1_ (36 ± 5 nm) despite affinities of 0.5 ± 0.1 μm and 4 ± 0.6 nm, respectively (*Bs* aptamer). These findings suggest that the region of **1** situated near C15 would be a good candidate for synthetic efforts to improve the potency for downregulating gene expression. Our co-crystal structures are the starting point for developing additional compounds that target the bacteria-specific Q biosynthetic pathway.

Although **1**, **2**, and **3** have similar binding affinities for the *Tt* and *Bs* aptamers, they do not have identical activity in transcription termination with the *Ss* riboswitch. This difference in activity may be due to subtle differences in binding modes to the RNA, reflected in the lack of a hydrogen bond to G5 and the altered pose of ligand **3**. Importantly, the synthetic ligand has considerably decreased solubility at concentrations needed for transcription termination, which may also play a role in decreased function. Although it has been previously demonstrated that binding affinity is not the sole parameter that governs the activity of RNA-binding compounds^48^, these observations are often not accompanied by mechanistic or structural rationalization. Our work demonstrates that subtle alterations in the mode of recognition of ligands may also have a role in the ability of a compound to induce conformational or functional effects. This work highlights a challenge in developing ligands for functional RNAs, namely that affinity is not the only property that governs the activity of a compound. Finally, the work described herein illustrates the role structure has in understanding the behavior of small synthetic compounds that bind to and modulate the function of complex RNAs.

## Methods

### General RNA methods

To avoid RNase contamination, all buffers were prepared with diethyl pyrocarbonate-treated water and all surfaces and equipment were decontaminated with RNaseZap (Ambion) prior to RNA handling. Deprotected and high-performance liquid chromatography purified oligonucleotides were purchased from Dharmacon (ThermoFisher) or IDT DNA. Sequences of the RNA oligonucleotides used for biochemical and biophysical experiments can be found in Supplementary Table 1.

### SMM screening

SMM slides were prepared according to established procedures^36,38^. In brief, γ-aminopropyl silane microscope slides (Corning) were functionalized with an Fmoc-protected amino polyethylene glycol spacer (Fmoc-8-amino-3,6-dioxaoctanoic acid) in *N,N*-dimethylformamide (DMF). Following piperidine deprotection, 1,6-diisocyanatohexane was coupled to the surface to provide isocyanate-functionalized microarray slides for immobilization of small molecule library members. A total of 26,227 small molecules (10 mm in DMSO) containing at least one primary or secondary alcohol or amine were purchased from commercial vendors including ChemBridge and ChemDiv. The libraries were printed on seven array slides containing ~ 3840 distinct molecules printed in duplicate, in addition to dyes and controls used for quality-control validation. The arrays were exposed to pyridine vapor to facilitate covalent attachment to the isocyanate-functionalized slides. Slides were then incubated with a 1:20 polyethylene glycol:DMF (v/v) solution to quench unreacted isocyanates. The 5′-Cy5-*Bs*PreQ_1_-RS RNA was dissolved in 50 mm Tris, 100 mm KCl, 1 mm MgCl_2_, pH 7.5, diluted to 5 µm, and was annealed by heating to 75 °C for 5 min, followed by slowly cooling to room temperature for 30 min. The annealed RNA was then further diluted to 1 µm in 50 mm Tris, 100 mm KCl, 1 mm MgCl_2_, pH 7.5 for screening. Printed microarray slides were incubated with the RNA at a concentration of 1 µm for 2 h using a LifterSlip (Electron Microscopy Sciences). Following incubation, slides were washed three times with 4 mL of 50 mm Tris, 100 mm KCl, 1 mm MgCl_2_, pH 7.5 buffer for 2 min in a four-well Nunc plate, and the slides were dried by centrifugation for 2 min at 4000 × g. Slides were imaged for fluorescence (650 nm excitation, 670 nm emission) on an Innopsys Innoscan 1100 AL Microarray Scanner with a resolution of 5 μm. The scanned image was aligned with the corresponding GenePix Array List (GAL) file to identify individual features. Hits were determined based on signal-to-noise ratio (SNR), defined as (mean foreground−mean background)/standard deviation of background, and *Z*-score, defined as: *Z* = (Mean SNR635compound—Mean SNR635library)/(SD SNR635library) with the following criteria: (a) coefficient of variance of duplicate spots < 100, (b) Average Z-score for a compound > 3, (c) [(*Z*-score_RNA incubated_)—(*Z*-score_Control Array_)]/*Z*-score_Control Array_) > 3, (d) no activity with any other nucleic acid structures screened in parallel. Other Cy5-labeled riboswitches screened in parallel included TPP and SAM-II, which were screened using the same method described above for the PreQ_1_ riboswitch. Hits were further validated by visual inspection of array images and compounds for further study were purchased from original suppliers.

### General ligand observed NMR methods

All NMR spectra were recorded at 293 K on a Bruker AVANCE III 500 MHz spectrometer equipped with a TCI cryoprobe. NMR buffer was composed of 50 mm Tris-*d*_11_, pH 7.5, 100 mm KCl, 1 mm MgCl_2_ in 100% UltraPure distilled H_2_O (Invitrogen). Subsequently prepared NMR samples contained 10% D_2_O and 5% DMSO-*d*_6_ to improve compound solubility. All compounds were first dissolved to a 10 mm stock concentration in 100% DMSO-*d*_6_ prior to NMR sample preparation. Purchased RNA was buffer exchanged (3 kDa MWCO spin column, EMD Millipore) into the NMR buffer prior to use. For WaterLOGSY and CPMG experiments, a reference 1D-^1^H spectrum was collected for each sample using the “zgesgp” excitation sculpting water suppression pulse sequence from Bruker, with 128 scans. All data was processed and visualized with MestReNova software (Version 8.1.2-11880).

### WaterLOGSY NMR

*Bs*PreQ_1_-RS and *Tt*PreQ_1_-RS RNA were buffer exchanged into 50 mm Tris-*d*_11_, pH 7.5, 100 mm KCl, 1 mm MgCl_2_ in UltraPure distilled H_2_O using centrifugal filtration (3 kDa MWCO, EMD Millipore) and were annealed by heating to 75 °C for 5 min, followed by slowly cooling to room temperature for 30 min. NMR samples containing each SMM compound at a concentration of 375 µm and a negative control *N*-methyl-l-valine (Chem-Impex-International) at 375 µm were prepared in 50 mm Tris-*d*_11_, pH 7.5, 100 mm KCl, 1 mm MgCl_2_ in UltraPure distilled H_2_O containing 10% D_2_O and a final concentration of 5% DMSO-*d*_6_. For each compound, samples were prepared with and without each RNA at a concentration of 15 µm. Samples were incubated on ice for 10 min, degassed at 20 °C for 15 min, and transferred to a Shigemi NMR tube. A reference 1D-^1^H and 1D WaterLOGSY spectra with and without RNA were recorded. NMR spectra were recorded at 293 K on a Bruker AVANCE III 500 MHz spectrometer equipped with TCI cryoprobe^43^.

### CPMG NMR

*Bs*PreQ_1_-RS and *Tt*PreQ_1_-RS RNA were buffer exchanged into 50 mm Tris-*d*_11_, pH 7.5, 100 mm KCl, 1 mm MgCl_2_ in UltraPure distilled H_2_O using centrifugal filtration (3 kDa MWCO, EMD Millipore) and were annealed by heating to 75 °C for 5 min, followed by slowly cooling to room temperature for 30 min. Samples of **1** (300 μm) with or without each RNA (3 μm) were prepared in 50 mm Tris-*d*_11_, pH 7.5, 100 mm KCl, 1 mm MgCl_2_ in UltraPure distilled H_2_O containing *N*-methyl-l-valine (300 μm), 10% D_2_O, and 5% DMSO-*d*_6_. Samples were incubated on ice for 10 min, degassed at 20 °C for 15 min, and transferred to a Shigemi NMR tube. NMR spectra were acquired at 298 K on a Bruker AVANCE III 500 MHz spectrometer fitted with a TCI Cryoprobe with Z-gradient. CPMG data sets with Presaturation Water Suppression (cpmgpr1d) were acquired using a 200 ms T_2_ relaxation filter (d_2_0=0.001 s, L4=200) with a 10-second relaxation delay. O1 was set to 2349.67 Hz, RG to 203, and data sets were acquired with 64 scans. The spectra of the sample with and without RNA were aligned and scaled so that the peak heights of the internal standard *N*-methyl-l-valine were equivalent. Significantly attenuated ligand peaks (≥ 20%) in the presence of RNA were considered indicative of small molecule binding^45^.

### Fluorescence intensity assay

Fluorescence titrations were performed using either a 5′-Cy5-*Bs*PreQ1-RS or AF647-*Tt*PreQ1-RS. Each RNA was diluted into 50 mm Tris, 100 mm KCl, 1 mm MgCl_2_, pH 7.5 to a concentration of 10 µm and was annealed by heating to 75 °C for 5 min, followed by slowly cooling to room temperature for 30 min. Small molecule solutions were prepared as serial dilutions in 10% DMSO in buffer. In a cuvette, RNA was diluted to a final concentration of 50 nm in 50 mm Tris, 100 mm KCl, 1 mm MgCl_2_, pH 7.5 buffer containing 10% DMSO. Small molecules were titrated to final concentrations ranging from 0 to 77.4 μm maintaining a 10% final DMSO concentration. After delivery of the small molecule, the sample was allowed to equilibrate for 8 min at room temperature. The fluorescence emission spectrum was then measured at room temperature using a Photon Technology International, Inc. QuantaMaster 600^TM^ Spectrofluorometer equipped with Felix GX 4.2.2 software. Fluorescence was recorded at an excitation wavelength of 649 nm and an emission of 655–800 nm. Total area under the peak from 655–700 nm was quantified and was then normalized to the values obtained for RNA incubated with a DMSO control. Normalized fluorescence for three independent replicates were averaged and plotted against small molecule concentration. *K*_D_ values were determined using a single site-binding model.

### Inherent ligand fluorescence titration

Fluorescence titrations were performed using either unlabeled *Bs*PreQ1-RS or *Tt*PreQ_1_-RS. Each RNA was resuspended to a concentration of ~ 500 µm in 50 mm Tris, pH 7.5, 100 mm KCl, 1 mm MgCl_2_, and annealed by heating to 75 °C for 5 min, followed by slowly cooling to room temperature for 30 min. Serial dilutions of each RNA were prepared in 50 mm Tris, pH 7.5, 100 mm KCl, 1 mm MgCl_2_. Inherent fluorescence titrations were performed with compound **1** at a final concentration of 500 nm in 50 mm Tris, pH 7.5, 100 mm KCl, 1 mm MgCl_2_ with 5% DMSO. For *Bs*PreQ_1_-RS, in a black 384-well plate, **1** was diluted to a final concentration of 500 nm in buffer with a 5% final DMSO concentration. RNA was added to final concentrations ranging from 0–50 μm in triplicate, the plate was centrifuged (1000 rpm, 2 min), and samples were allowed to incubate for 30 min at room temperature with shaking. The fluorescence intensity was then measured on a Synergy Mx microplate reader (BioTek) at an excitation wavelength of 300 nm and an emission wavelength of 340 nm (gain = 120). The fluorescence intensity was then normalized to the values obtained for **1** in the absence of RNA and was plotted against RNA concentration. The dissociation constants were determined using a single site binding model.

For *Tt*PreQ_1_-RS, in a cuvette, **1** was diluted to a final concentration of 500 nm in 50 mm Tris, pH 7.5, 100 mm KCl, 1 mm MgCl_2_ buffer with a 5% final DMSO concentration. RNA was titrated to final concentrations ranging from 0 to 7 μm and the sample was allowed to equilibrate for 8 min at room temperature after each addition. The fluorescence emission spectrum was then measured at room temperature using a Photon Technology International, Inc. QuantaMaster 600^TM^ Spectrofluorometer equipped with Felix GX 4.2.2 software. Fluorescence was recorded at an excitation wavelength of 300 nm and an emission of 320-460 nm. Total area under the peak from 320-380 nm was quantified and was then normalized to the values obtained for **1** in the absence of RNA. Normalized fluorescence for three independent replicates were averaged and plotted against RNA concentration. *K*_D_ values were determined using a single site binding model.

### Analysis of Mg^2+^-induced in-line cleavage

This procedure was adapted from a related procedure that used radiolabeled oligonucleotides^30^. 5′-AF647-*Bs*PreQ_1_-RS or 5′-AF647-*Tt*PreQ_1_-RS RNA was diluted into 50 mm Tris, 100 mm KCl, pH 7.5 to a concentration of 10 µm and were annealed by heating to 75 °C for 5 min, followed by cooling on ice for 10 min. The annealed RNA was incubated at a final concentration of 1 μm in 50 mm Tris, pH 8.3, 10 mm KCl buffer with either a DMSO control (5% final concentration) or compound **1** at a concentration of 0.1, 1, 3, 10, 30, 100, or 300 μm. For 5′-AF647-*Bs*PreQ_1_-RS, MgCl_2_ was added at a final concentration of 2 mm, and the reactions were incubated at room temperature in darkness for 40 hrs. For 5′-AF647-*Tt*PreQ_1_-RS, MgCl_2_ was added at a final concentration of 2 mm, and the reactions were incubated at room temperature in darkness for 72 h to reach the desired level of cleavage. Alkaline hydrolysis was performed in 50 mm NaHCO_3_, pH 9.0 at 95 °C for 5 min. Ribonuclease T1 digestion was carried out with 0.1 U of ribonuclease T1 (Ambion) in 20 mm Tris, pH 7.5, 50 mm NaCl, 0.1 mm MgCl_2_ at room temperature for 20 min. The RNase T1 reaction was stopped by adding 0.2 volumes of 5 mm EDTA. Equal volumes of loading buffer containing 7 m urea, 1 × Tris-borate-EDTA (TBE), and 0.01% direct red dye was added to each reaction, and the samples were heated to 95 °C for 5 min. 10 µL of each sample was analyzed by electrophoresis on a denaturing polyacrylamide sequencing gel (20% polyacrylamide, 19:1 crosslinking, 7 m urea) at 60 W, 45 °C for 3.5 hrs. The gel was visualized by fluorescence of the 5′-Cy5 label (630 nm excitation, 670 emission) with Typhoon FLA 9500 Phosphorimager (GE Healthcare Life Sciences) and was analyzed with ImageQuant software.

### X-ray crystallography RNA preparation

Sequences of the RNAs employed in this study are based on *Tt*PreQ1 riboswitch aptamer domain and listed in Supplementary Table 1. RNAs were purchased from Dharmacon and deprotected according to the manufacturer’s instructions. After lyophilization, RNAs were dissolved in water and stored at − 25 °C.

### Crystallization and diffraction data collection

RNA solutions containing 11.8 mm sodium cacodylate (pH 7.0) were heated at 65 °C for 2 min. MgCl_2_ and synthetic compound were then added to the final concentrations of 10 mm and 0.5 mm, respectively. The solutions were incubated at 65 °C for 3 min, and then cooled down gradually to the room temperature. *Tt*PreQ_1_ riboswitch was crystallized by the hanging-drop vapor diffusion method at 21 °C, under conditions containing 5–15 mm Mg acetate, 50 mm MES (pH 5.6), and 2.3–2.7 m ammonium sulfate (Condition I) and containing 0.1–0.3 m potassium sodium tartrate, 100 mm sodium citrate (pH 5.6), and 2.0–2.8 m ammonium sulfate (Condition II). Hanging drops were prepared by mixing 1 μL of the RNA solution (0.38 mm RNA in 10 mm sodium cacodylate, pH 7.0, 10 mm MgCl_2_, and 0.5 mm synthetic compound) with 1 μL of the reservoir solution and were equilibrated against 400 μL of reservoir solution. The RNA crystals grew within 2 weeks to maximum dimensions of 300 × 100 × 100 μm^3^. For data collection, the RNA crystals were transferred to the cryoprotectant solutions containing 10 mm Mg acetate, 27.5 mm MES (pH 5.6), and 2.15 M lithium sulfate for the crystals obtained with the Condition I, and 60 mm potassium sodium tartrate, 25 mm sodium citrate (pH 5.6), and 2.15 M lithium sulfate for the crystals under the Condition II. The crystals were mounted in a nylon loop and flash frozen by plunging into liquid nitrogen. X-ray diffraction data were collected at the beamlines 5.0.1 and 5.0.2 of the Advanced Light Source (ALS), Lawrence Berkeley National Laboratory. Diffraction data were integrated and scaled with the program DIALS^49^. Data processing statistics are summarized in Supplementary Table 7.

### Structure determination and refinement

The structures were solved by the MR method using the previously determined *T. tengcongensis* PreQ_1_ aptamer structure (PDB ID: 3Q51 [10.2210/pdb3Q51/pdb]) as a search model with the program PHASER^50^. The solutions were subjected to simulated annealing, energy minimization, restrained isotropic B-factor, and TLS refinement with PHENIX^51^, and the resulting electron-density maps revealed the locations of the small molecules. Iterative cycles of refinement and manual rebuilding^52^ produced the current co-crystal structures of ab13_14-**1**, ab13_14_15-**1**, ab13_14-**2**, ab13_14_15-**3**, and ab13_14-PreQ_1_ with *R*_free_ of 20.5, 20.5, 20.3, 24.8, and 21.0% at 1.80, 1.80, 1.94, 2.56, and 1.69 Å resolution, respectively. Refinement statistics are summarized in Supplementary Table 7. Molecular graphics were produced with PyMol (http://www.pymol.org/). Stereo images are provided in Supplementary Figure 12 and 13.

### Single-round transcription termination assay

The transcription termination assays were carried out according to established protocols^53,54^ with several modifications. The DNA plasmid containing *λ*_PR_ promoter and 26-nt C-less sequence followed by the *Staphylococcus saprophyticus* PreQ_1_ riboswitch and its downstream sequence cloned into pIDTSMART-AMP was purchased from Integrated DNA Technologies. The DNA template was amplified by PCR from the plasmid using forward and reverse primers (Supplementary Table 6), and then was gel-extracted after agarose gel electrophoresis for purification. Halted transcription complexes were prepared in a solution containing 1 µm GTP, 5 µm ATP, 5 µm UTP, 100 µm ApU, [α-^32^P] GTP, 75 nm DNA template, 0.0167 U/µL *Escherichia coli* RNA polymerase holoenzyme (New England BioLabs) in 1 × transcription buffer (20 mm Tris-HCl, pH 8.0, 2 mm NaCl, 1 mm MgCl_2_, 4% glycerol, 0.1 mm DTT, and 0.1 mm EDTA), and incubated at 37 °C for 15 min. A DNA oligonucleotide complementary to the 26-nt C-less sequence was added to the reactions at 1.1 µm final concentration in 1 × transcription buffer and incubated at room temperature for 5 min, in order to prevent undesired non-specific interactions between the 26-nt C-less sequence and riboswitch. Elongation was restarted by combining 9 µL of halted transcription complex, 3 µL of the compound (0–25 mm compound and 25% DMSO in 1 × transcription buffer), and 3 µL of NTPs mix (200 µm ATP, 200 µm CTP, 200 µm GTP, 200 µm UTP, 100 µg/mL heparin, and 250 mm KCl in 1 × transcription buffer), and incubated at 37 °C for 20 min. To remove the DNA template, 0.5 U of RQ1 RNase-Free DNase (Promega) was added to the reactions and incubated at 37 °C for 10 min. The reactions were stopped by adding equal volume of loading dye (8 m urea, 20% sucrose, 0.05% bromophenol blue, and 0.05% xylene cyanol in 2 × TBE). The reaction mixture was separated by 8% denaturing PAGE and visualized by phosphorimager. Full gel images are provided as Supplementary Figures. The band intensity was analyzed by ImageQuant software (GE Healthcare). Termination efficiency was calculated with dividing the intensity for the terminated RNA band by those for the total (terminated and antiterminated) RNAs. The sequences of the DNA oligonucleotides used in the transcription termination assay are summarized in Supplementary Table 5.

### Synthetic procedures and characterization

Chemical synthesis and compound characterization are provided in the Supplementary Methods.

### Reporting summary

Further information on experimental design is available in the Nature Research Reporting Summary linked to this article.

## Supplementary information

Supplementary Information

Reporting Summary

Source Data

## Data Availability

Atomic coordinates and the structure factors of the co-crystal structures of ab13_14-**1**, ab13_14_15-**1**, ab13_14-**2**, ab13_14_15-**3**, and ab13_14-PreQ_1_ have been deposited in the Protein Data Bank, under the accession codes 6E1S, 6E1T, 6E1U, 6E1V and 6E1W, respectively. The source data underlying Figs. 3A–B, 5B, 7B and Supplementary Figs 2, 3, 5, 7B, and 10 are provided as a Source Data file. All other relevant data are available from the authors upon request.
