# Supplementary material for: Synthetic ligands for PreQ1 riboswitches provide structural and mechanistic insights into targeting RNA tertiary structure
Source: Nat Commun. 2019 Apr 2;10:1501. doi: 10.1038/s41467-019-09493-3 (PMC6445138; doi:10.1038/s41467-019-09493-3)
Supplement: Supplementary file 1 — Supplementary Information [file 41467_2019_9493_MOESM1_ESM.pdf]

Synthetic Ligands for $PreQ_1$ Riboswitches Provide Structural and Mechanistic Insights into Targeting RNA Tertiary Structure

Colleen M. Connelly,[1¶] Tomoyuki Numata,[2,3¶] Robert E. Boer,[1] Michelle H. Moon,[1] Ranu S. Sinniah,[1] Joseph J. Barchi,[1] Adrian R. Ferré-D'Amaré,[2] and John S. Schneekloth, Jr.[1,]*

Supplementary Information

Supplementary Methods:

General Chemistry Methods.

Unless otherwise noted, all chemical reagents were obtained from commercial suppliers and used without further purification. Anhydrous CH_3CN, DMF, and THF were obtained from GlassContour Solvent Systems and were dried over alumina under an argon atmosphere. Solvents were removed using a Buchi rotary evaporator under reduced pressure. Flash column chromatography was performed using a Teledyne ISCO CombiFlash Rf automated chromatography system.

^{1}H and ^{13}C NMR spectra were recorded on Varian and Bruker spectrometers (at 500 MHz or at 125 MHz, respectively) and are reported relative to deuterated solvent signals. Data for ^{1}H NMR spectra are reported as follows: chemical shift (δ ppm), multiplicity (s = singlet, d = doublet, t = triplet, q = quartet, m = multiplet, br = broad, app = apparent), coupling constants (Hz), and integration. Data for ^{13}C NMR spectra are reported in terms of chemical shift.

High resolution mass spectrometry data were acquired on an Agilent 6520 Accurate-Mass Q-TOF LC/MS System, (Agilent Technologies, Inc.) equipped with a dual electro-spray source, operated in the positive-ion mode. Separation was performed on Zorbax 300SB-C18 Poroshell column (2.1 mm x 150 mm; particle size 5 μm). The analytes were eluted using a water/acetonitrile gradient with 0.1% formic acid. Data were acquired at high resolution (1,700 *m/z*), 4 GHz. To maintain mass accuracy during the run time, an internal mass calibration sample was infused continuously during the LC/MS runs. Data acquisition and analysis were performed using MassHunter Workstation Data Software, LCMS Data Acquisition (version B.06.01) and Qualitative Analysis (version B.07.00).

Chemistry Experimental Procedures:

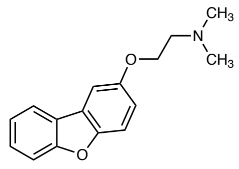

Dibenzofuran 2: To a solution 2-(dibenzo[b,d]furan-2-yloxy)ethanamine (20.0 mg, 0.0649 mmol) in DMF/DCE (1:1, 2.0 mL) was added formaldehyde solution (37 wt% in H_2O, 33 μL, 0.440 mmol). $Na(OAc)_3BH$ (93.0 mg, 0.440 mmol) was added and the reaction was stirred at room temperature for 3 h. The reaction mixture was concentrated *in vacuo* and the resulting residue was purified by Isco Column Chromatography (0-25% MeOH in CH_2Cl_2) to produce 17 mg (76%) of **2** as an off white solid. ^{1}H NMR (500 MHz, DMSO-d_6) δ 8.12 (dt, *J* = 7.6, 1.0 Hz, 1H), 7.74 (d, *J* = 2.6 Hz, 1H), 7.65 (dt, *J* = 8.3, 0.8 Hz, 1H), 7.59 (d, *J* = 8.9 Hz, 1H), 7.50 (ddd, *J* = 8.4, 7.2, 1.4 Hz, 1H), 7.38 (td, *J* = 7.5, 0.9 Hz, 1H), 7.09 (dd, *J* = 8.9, 2.7 Hz, 1H),

4.15 (t, J = 5.8 Hz, 2H), 2.68 (t, J = 5.8 Hz, 2H), 2.25 (s, 6H). ^{13}C NMR (125 MHz, DMSO-d_6) δ 156.1, 154.8, 150.0, 127.5, 124.2, 123.9, 122.8, 121.2, 115.9, 112.1, 111.7, 105.1, 66.4, 57.6, 45.4 (2C). HRMS: (ESI+) m/z calculated for $C_{16}H_{18}NO_2$ $[M+H]^+$: 256.1332, found: 256.1324. Spectra are provided in Supplementary Figure 14.

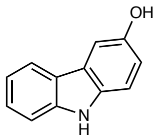

Carbazole 4: To a flame-dried flask equipped with stir-bar was added 3-methoxy-9H-carbazole (140 mg, 0.710 mmol) and CH_2Cl_2 (25 mL). A solution of BBr_3 (1.0 M in CH_2Cl_2, 2.13 mL, 2.13 mmol) was added dropwise slowly and the reaction stirred at room temperature for 3 h. The reaction was cooled to 0 °C, treated with MeOH (1 mL), and stirred for 5 min. The reaction mixture was then diluted with H_2O and the aqueous layer extracted with CH_2Cl_2 (3 x 20 mL). The combined organic layers were dried ($MgSO_4$), filtered, and concentrated *in vacuo*. The resulting residue was purified by Isco flash column chromatography (0-100% EtOAc in hexanes) to afford 117 mg (90%) of carbazole **4** as a light brown solid. The spectral data matched reported values.[1]

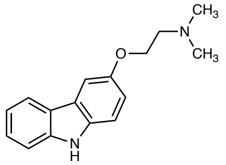

Carbazole 3: To a solution of carbazole **4** (10.0 mg, 0.0546 mmol) in CH_3CN (1 mL) was added K_2CO_3 (9.0 mg, 0.0655 mmol) and 2-Chloro-*N*,*N*-dimethylethylamine hydrochloride (9.4 mg, 0.0655 mmol). The reaction was heated to reflux and stirred for 16 h. After cooling to room temperature, the reaction was concentrated, and the resulting residue dissolved in CH_2Cl_2 and H_2O. The aqueous layer was extracted with CH_2Cl_2 (3 x 10 mL) and the organics were washed with H_2O (10 mL) and brine (10 mL), dried (Na_2SO_4), filtered and concentrated *in vacuo*. The resulting residue was purified by Isco flash column chromatography (0-25% MeOH in CH_2Cl_2) to afford 8 mg (60%) of **3** as a light brown solid. ^{1}H NMR (500 MHz, DMSO-d_6) δ 11.03 (s, 1H), 8.08 (d, J = 7.7 Hz, 1H), 7.70 (d, J = 2.5 Hz, 1H), 7.43 (d, J = 8.1 Hz, 1H), 7.37 (d, J = 8.7 Hz, 1H), 7.34 (ddd, J = 8.2, 7.0, 1.2 Hz, 1H), 7.13-7.08 (m, 1H), 7.02 (dd, J = 8.7, 2.5 Hz, 1H), 4.15 (t, J = 5.8 Hz, 2H), 2.75 (t, J = 5.8 Hz, 2H), 2.31 (s, 6H). ^{13}C NMR (125 MHz, DMSO-d_6) δ 152.0, 140.4, 134.6, 125.4, 122.8, 122.4, 120. 3, 118.0, 115.2, 111.5, 110.9, 104.1, 66.2, 57.8, 45.4 (2C). HRMS: (ESI+) m/z calculated for $C_{16}H_{19}N_2O$ $[M+H]^+$: 255.1492, found: 255.1483. Spectra are provided in Supplementary Figure 15.

Supplementary Table 1. Sequences of riboswitch oligonucleotides.

Name	Sequence (5′ – 3′)	Vendor	Experiment
5′-Cy5-*Bs*PreQ$_1$-RS	Cy5-AGA GGU UCU AGC UAC ACC CUC UAU AAA AAA CUA A	Dharmacon	SMM, FIA
5′-Cy5-SAM-II-RS	Cy5-UCG CGC UGA UUU AAC CGU AUU GCA AGC GCG UGA UAA AUG UAG CUA AAA AGG G	Dharmacon	SMM
5′-Cy5-TPP-RS	Cy5-CAG UAC UCG GGG UGC CCU UCU GCG UGA AGG CUG AGA AAU ACC CGU AUC ACC UGA UCU GGA UAA UGC CAG CGU AGG GAA GUG CUG	Dharmacon	SMM
*Bs*PreQ$_1$-RS (unlabeled)	AGA GGU UCU AGC UAC ACC CUC UAU AAA AAA CUA A	Dharmacon	Ligand Fluorescence Titration, NMR
5′-Biotin-*Bs*PreQ$_1$-RS	Bi-GGA GAG GUU CUA GCU ACA CCC UCU AUA AAA AAC UAA	Dharmacon	SPR
5′-AF647-*Bs*PreQ$_1$-RS	AlexaFluor647*-GGA GAG GUU CUA GCU ACA CCC UCU AUA AAA AAC UAA	IDT DNA	In-line Probing
5′-AF647-*Tt*PreQ$_1$-RS	AlexaFluor647*-CUG GGU CGC AGU AAC CCC AGU UAA CAA AAC AAG	IDT DNA	FIA, In-line Probing
*Tt*PreQ$_1$-RS (unlabeled)	CUG GGU CGC AGU AAC CCC AGU UAA CAA AAC AAG	Dharmacon	Ligand Fluorescence Titration, NMR
5′-Biotin-*Tt*PreQ$_1$-RS	Bi-CUG GGU CGC AGU AAC CCC AGU UAA CAA AAC AAG	Dharmacon	SPR
*Ss*PreQ$_1$-RS (unlabeled)	AGA GGU UCC UAG CUG AUA CCC UCU AUA AAA AAC UA	Dharmacon	NMR
ab13_14	CUG GGU CGC AGU NNC CCC AGU UAA CAA AAC AAG	Dharmacon	Crystallography
ab13_14_15	CUG GGU CGC AGU NNN CCC AGU UAA CAA AAC AAG	Dharmacon	Crystallography

N shows an abasic site of the RNA
*5’-modification is accomplished with AlexaFluor647-NHS ester

Supplementary Table 2. Selected sequences of RNA and DNA oligonucleotides screened by SMM (Figure 1C).

Oligonucleotide	Name	Structure	Sequence (5′ – 3′)
1	Pre-miR-21[2]	RNA hairpin	AlexaFluor647*–UAGCUUAUCAGACUGAUGUUGACUGUUGAAUCUCAUGGCAACACCAGUCGAUGGGCUGUC
2	Pre-miR-17[3]	RNA hairpin	AlexaFluor647*–CAAAGUGCUUACAGUGCAGGUAGUGAUAUGUGCAUCUACUGCAGUGAAGGCACUUGUAG
3	Pre-miR-31[3]	RNA hairpin	AlexaFluor647*–AGGCAAGAUGCUGGCAUAGCUGUUGAACUGGGAACCUGCUAUGCCAACAUAUUGCCAUC
8	HIV RRE IIB[4]	RNA hairpin	AlexaFluor647*–UGGGCGCAGUGUCAUUGACGCUGACGGUACA
14	Malat1[5]	RNA triple helix	AlexaFluor647*–AAAGGUUUUUCUUUUCCUGAGAAAUUUCUCAGGUUUUGCUUUUUAAAAAAAAAGCAAAA
15	Zika Ns5[6]	RNA G4	AlexaFluor647*–GUGGAGGUGGGACGGGAGAGACUCUGGGAGA
16	Zika 3'UTR[6]	RNA G4	AlexaFluor647*–GCGGCGGCCGGUGUGGGGAA
17	NRAS[7]	RNA G4	AlexaFluor647*–UGUGGGAGGGGCGGGUCUGGG
18	TERRA[8]	RNA G4	AlexaFluor647*–GGGUUAGGGU
19	EWSR1[9]	RNA G4	AlexaFluor647*–GGGGCAGGGGAAGAGGGGG
20	AKTIP[10]	RNA G4	AlexaFluor647*–GGGGUGGGGCGGGGCGGG
22	MYB[11]	DNA G4	AlexaFluor647*–GGAGGAGGAGGTCACGGAGGAGGAGGAGAAGGAGGAGGAGGA
23	MYC[12]	DNA G4	AlexaFluor647*–AGGGTGGGGAGGGTGGGG
24	KRAS[11]	DNA G4	AlexaFluor647*–AGGGCGGTGTGGGAAGAGGGAAGAGGGGGAGGCA
26	VEGF[11]	DNA G4	AlexaFluor647*–CGGGGCGGGCCGGGGGCGGGGT
27	RB1[11]	DNA G4	AlexaFluor647*–CGGGGGGTTTTGGGCGG
28	BCL2[13]	DNA G4	AlexaFluor647*–AGGGGCGGGCGCGGGAGGAAGGGGGCGGGA
29	cKIT[14]	DNA G4	AlexaFluor647*–AGGGAGGGCGCTGGGAGGAGGG

*5’-modification is accomplished with AlexaFluor647-NHS ester

Supplementary Table 3. Commercial Supplier IDs for 86 candidate compounds from the SMM.*

Supplier Product ID				
9188721 (**1**)	5224153	2810-4227	8019-9983	NSC79486
17311387 (**SI1**)	5269563	2810-4329	8020-0820	NSC142269
42028139 (**SI2**)	5325779	2810-4334	8020-2578	Tobramycin
9205277 (**SI3**)	6044923	3639-0494	8020-2683	Nedaplatin
9190966 (**SI4**)	6357130	4269-3090	8104-04926	BMS-536924 (IGF-1R Inhibitor)
5320336 (**SI5**)	6372845	4576-0108	8388-1007	KUC107871N-04 (p97 ATPase Inhibitor)
5718973 (**SI6**)	6802876	4788-0739	8388-1008	
8004-9540 (**SI7**)	9201127	5588-0118	C169-0255	
8007-1084 (**SI8**)	9202599	6233-2517	C201-0688	
8018-2852 (**SI9**)	1220123	6415-0035	C430-1098	
8020-1001 (**SI10**)	17922447	8005-0366	C749-0018	
8020-1052 (**SI11**)	18682916	8007-2266	C908-0403	
8104-04684 (**SI12**)	32903475	8010-4817	D245-0159	
C200-4138 (**SI13**)	33921774	8014-7935	D482-2054	
D063-1117 (**SI14**)	56240352	8016-5870	D704-1692	
D494-0506 (**SI15**)	0234-0037	8018-2758	D718-0691	
E595-0532 (**SI16**)	0632-0897	8018-2966	E470-0146	
8018-4095 (**SI17**)	1001-0003	8018-4095	E511-0764	
3738-4868 (**SI18**)	1134-0095	8018-7423	E679-0020	
4013-2178 (**SI19**)	2686-0069	8019-6726	NSC71881	

* For commercial suppliers, IDs with hyphenated numbers are available from ChemDiv (www.chemdiv.com), non-hyphenated numbers are from ChemBridge (www.chembridge.com), NSC numbers are from the NCI Developmental Therapeutics Program (https://dtp.cancer.gov/repositories/).

Supplementary Table 4. Selectivity of 20 purchased hit compounds across the $PreQ_1$, SAM-II and TPP riboswitches screened by SMM as determined by Z-score.

Supplier Product ID	Compound	Z-Score			
		Buffer	$PreQ_1$-RS	SAM-II-RS	TPP-RS
9188721	**1**	0.20	7.45	0.01	-0.44
17311387	**SI1**	-0.36	9.51	2.28	9.76
42028139	**SI2**	-1.21	15.53	5.47	4.74
9205277	**SI3**	-0.23	5.00	2.21	1.00
9190966	**SI4**	0.09	6.49	3.94	2.66
5320336	**SI5**	0.26	4.13	5.94	2.39
5718973	**SI6**	-0.45	3.44	1.08	-0.52
8004-9540	**SI7**	-0.39	5.48	1.44	1.11
8007-1084	**SI8**	1.19	8.53	2.41	2.51
8018-2852	**SI9**	0.12	8.32	0.76	0.72
8020-1001	**SI10**	0.76	8.89	1.48	1.99
8020-1052	**SI11**	0.14	5.61	1.97	1.31
8104-04684	**SI12**	-0.28	3.71	1.03	1.27
C200-4138	**SI13**	0.06	23.89	9.40	10.21
D063-1117	**SI14**	-0.11	22.28	4.14	8.74
D494-0506	**SI15**	-0.12	8.17	2.09	1.54
E595-0532	**SI16**	-0.11	14.47	6.13	6.4
8018-4095	**SI17**	0.04	5.52	0.68	1.59
3738-4868	**SI18**	0.69	3.25	7.09	1.93
4013-2178	**SI19**	0.60	19.98	19.71	11.84

Supplementary Table 5. Template DNA plasmids for transcription termination assays.

Name	Species	Sequence (5′ – 3′)	Cloned vector	RNA sequence (5′ – 3′)
Ss $PreQ_1$ TTA	*Staphylococcus saprophyticus*	TTGACTATTTTACC TCTGGCGGTGATAA TGGTTGCATGTAGT AAGGAGGTTGTATG GAAGACGAGAGGTT CCTAGCTGATACCC TCTATAAAAAACTA GACACATGTACAAC GTCTGTCTTTTTTA TAGAGATAGGCGTT TTTTTATGCGCTTA TCTAAACCCTGTAC CAGTTAGTTCGACT ATTTTTAAGGAGT	pIDTSMART-AMP	AUGUAGUAAGGAGGUUG UAUGGAAGACGAGAGGU UCCUAGCUGAUACCCUC UAUAAAAAACUAGACAC AUGUACAACGUCUGUCU UUUUUAUAGAGAUAGGC GUUUUUUUAUGCGCUUA UCUAAACCCUGUACCAG UUAGUUCGACUAUUUUU AAGGAGU
Bs $PreQ_1$ TTA	*Bacillus subtilis*	TTGACTATTTTACC TCTGGCGGTGATAA TGGTTGCATGTAGT AAGGAGGTTGTATG GAAGACGAGAGGTT CTAGCTACACCCTC TATAAAAAACTAAG GACGAGCTGTATCC TTGGATACGGCCTT TTTTATGTTTTTCT AGAGCACCTTCCGA AAAAAGGTGTTTTT TTGCGTGAATTAGC TGTAGC	pIDTSMART-AMP	AUGUAGUAAGGAGGUUG UAUGGAAGACGAGAGGU UCUAGCUACACCCUCUA UAAAAAACUAAGGACGA GCUGUAUCCUUGGAUAC GGCCUUUUUUAUGUUUU UCUAGAGCACCUUCCGA AAAAAGGUGUUUUUUUG CGUGAAUUAGCUGUAGC

Supplementary Table 6. Primers for transcription termination assays.

Primer name	Sequence (5′ – 3′)	Uses
Transcription forward primer	CAGTGAGTTGATTGCAGTCCAGTTACG CTG	PCR amplification from the *Ss* $PreQ_1$ TTA, *Bs* $PreQ_1$ TTA and *Bs* Apt *Bp* Ex $PreQ_1$ TTA plasmids
Transcription reverse primer for *Ss*	ACTCCTTAAAAATAGTCGAACTAACTG GTACAGGG	PCR amplification from the *Ss* $PreQ_1$ TTA
Transcription reverse primer for *Bs*	GCTACAGCTAATTCACGCAAAAAAACA CCTTTTTTCGG	PCR amplification from the *Bs* $PreQ_1$ TTA
Transcription reverse primer for *Bp*	TTTCTCAAAAGAAAAGACACCTTTACA TGTGTATGAGGTG	PCR amplification from the *Bs* Apt *Bp* Ex $PreQ_1$ TTA
26-nt C-less complementary sequence	TCTTCCATACAACCTCCTTACTACAT	Transcription termination assay to prevent undesired non-specific interactions between the 26-nt C-less sequence and riboswitch

Supplementary Table 7. Summary of data collection and refinement statistics.

	ab13_14-**1**	ab13_14_15-**1**	ab13_14-**2**	ab13_14_15-**3**	ab13_14-PreQ1
**Data collection**					
Space group	$P6_322$	$P6_322$	$P6_322$	$P6_322$	$P6_122$
Cell dimensions					
a, b, c (Å)	115.4, 115.4, 58.8	115.1, 115.1, 58.8	115.4, 115.4, 58.5	115.4, 115.4, 58.5	52.3, 52.3, 179.2
Wavelength (Å)	1.0	1.0	1.0	0.97741	1.0
Resolution (Å)	57.7-1.80 (1.85-1.80)	58.8-1.80 (1.85-1.80)	99.9-1.94 (1.99-1.94)	41.1-2.56 (2.67-2.56)	44.8-1.69 (1.72-1.69)
R_{merge}	0.080 (4.20)	0.081 (2.91)	0.094 (4.25)	0.090 (1.83)	0.036 (1.38)
$\langle I \rangle / \langle \sigma I \rangle$	15.7 (1.0)	16.9 (1.5)	15.0 (1.2)	29.1 (2.4)	44.4 (2.3)
Completeness (%)	100 (99.9)	99.8 (99.2)	100 (100)	100 (100)	99.6 (96.2)
Redundancy	25.4 (24.0)	25.4 (24.2)	25.6 (26.8)	37.7 (38.2)	33.2 (19.0)
**Refinement**					
Resolution (Å)	57.7-1.80	57.6-1.80	57.7-1.94	41.1-2.56	43.9-1.69
No. reflections	21,838	21,663	17,448	7,764	17,086
$R_{work}^{a}/R_{free}^{b}$	0.197/0.205	0.195/0.205	0.190/0.203	0.239/0.248	0.181/0.210
No. atoms					
RNA	674	674	674	674	675
Ligand	17	17	19	19	13
Ions	0	16	1	0	9
Water	66	83	50	2	96
B-factors (Å^2)					
RNA	49.8	43.9	59.5	81.2	48.5
Ligand	33.2	30.4	41.6	48.0	34.2
Ions	-	95.4	73.4	-	61.9
Water	50.3	47.2	56.1	51.2	55.0
R.m.s. deviations					
Bond lengths (Å)	0.005	0.005	0.004	0.005	0.005
Bond angles (º)	0.930	0.957	1.059	0.965	0.989
Estimated coordinate error (Å)	0.27	0.24	0.22	0.54	0.17
PDB ID	6E1S	6E1T	6E1U	6E1V	6E1W

The values in parentheses are for the outermost shell.

$^{a}R_{work} = \Sigma|F_o - F_c|/\Sigma F_o$ for reflections of working set.

$^{b}R_{free} = \Sigma|F_o - F_c|/\Sigma F_o$ for reflections of test set (5.0% of total reflections).

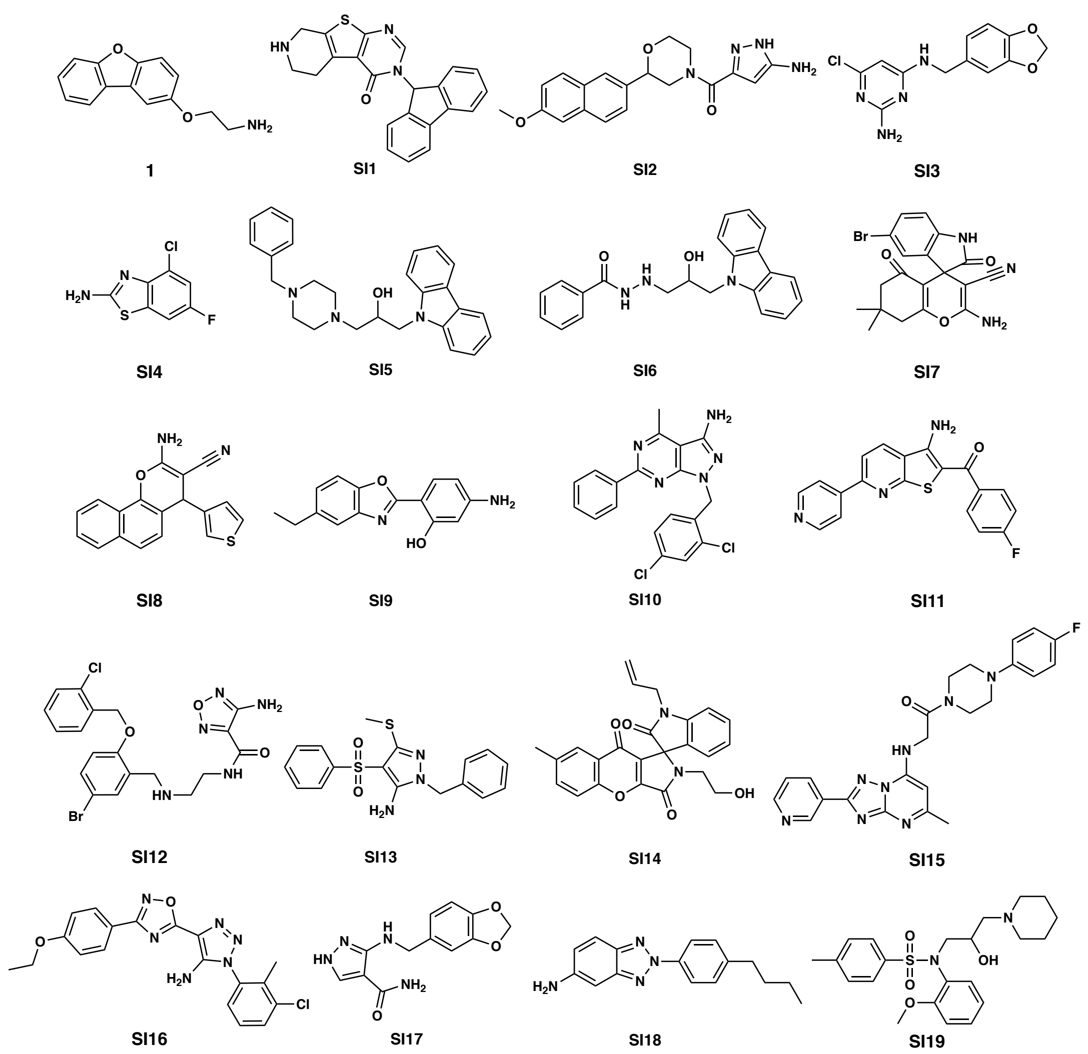

Supplementary Figure 1. Hit compounds for the 5′-Cy5-*Bs*PreQ$_1$-RS aptamer determined by SMM screening that were purchased for further analysis.

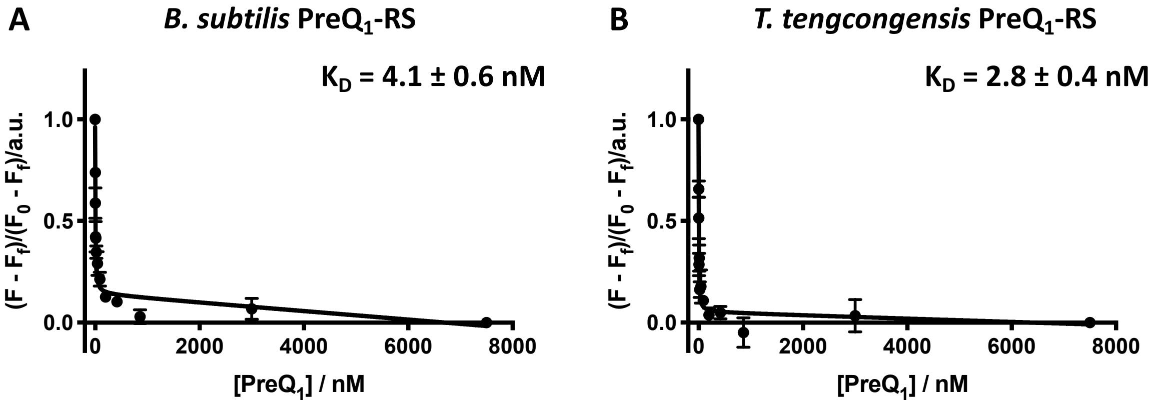

Supplementary Figure 2. Fluorescence intensity assay of 5′-Cy5-labeled *Bs*PreQ_1-RS **(A)** or 5′-AlexaFluor 647-labeled *Tt*PreQ_1-RS **(B)** RNA in the presence of increasing concentration of PreQ_1. Error bars indicate the standard deviation determined from three independent measurements.

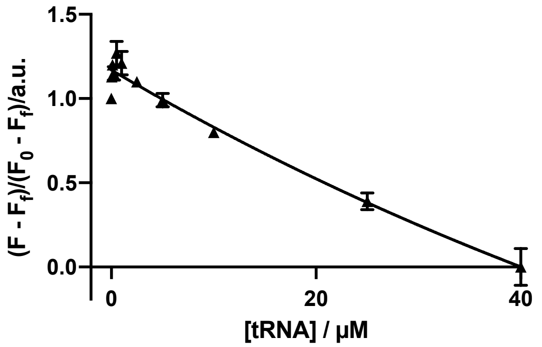

Supplementary Figure 3. Inherent fluorescence titration of **1** with increasing concentration of unlabeled yeast tRNA. Error bars indicate the standard deviation determined from three independent measurements.

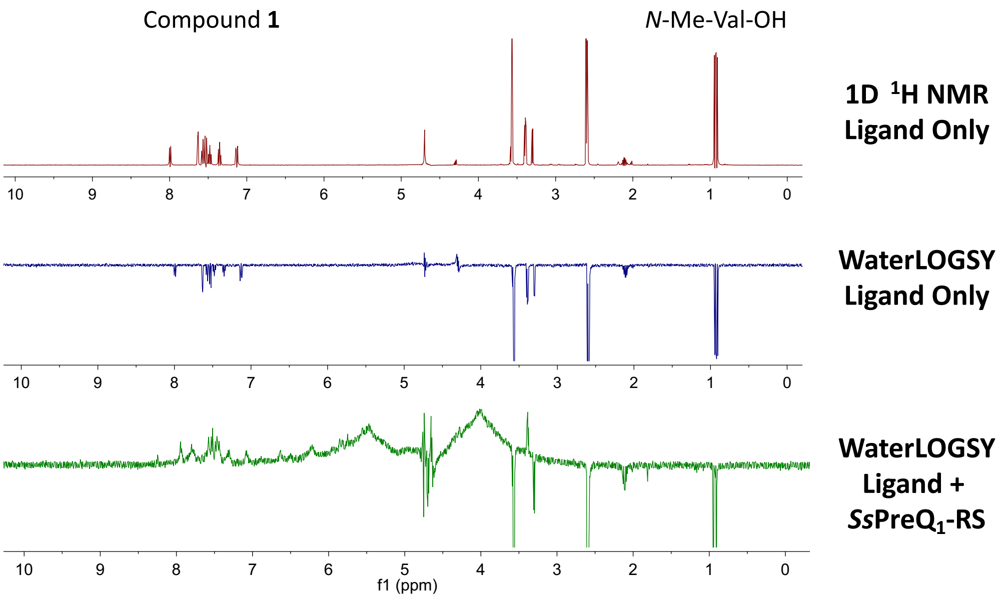

Supplementary Figure 4. NMR validation of compound **1** binding to the aptamer domain of the *Ss* $PreQ_1$ riboswitch. ^1H NMR of **1** and *N*-methyl-L-valine (non-binding control) (Top, red), WaterLOGSY NMR of **1** and *N*-methyl-L-valine in the absence (middle, blue) and presence (bottom, green) of unlabeled *Ss* $PreQ_1$ riboswitch aptamer.

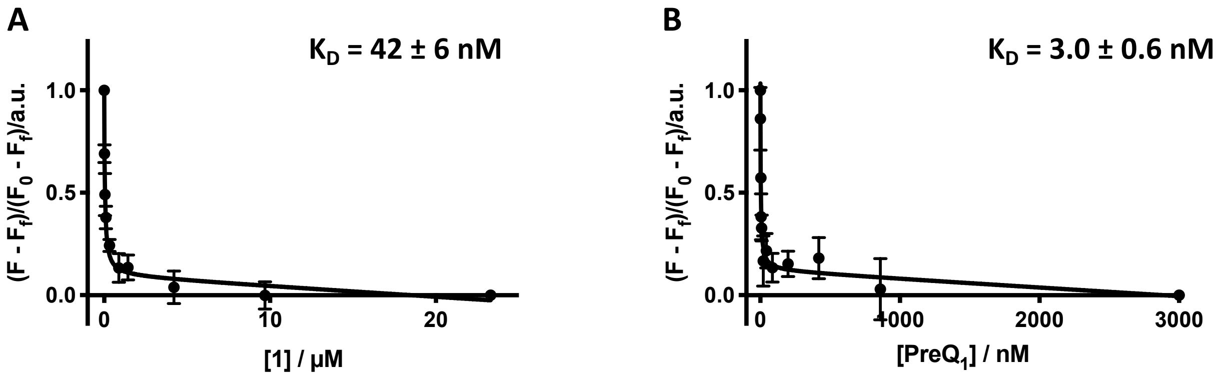

Supplementary Figure 5. Fluorescence intensity assay of 5′-AlexaFluor 647-labeled *Ss*PreQ$_1$-RS RNA in the presence of increasing concentration of **1 (A)** or PreQ$_1$ **(B)**. Error bars indicate the standard deviation determined from three independent measurements.

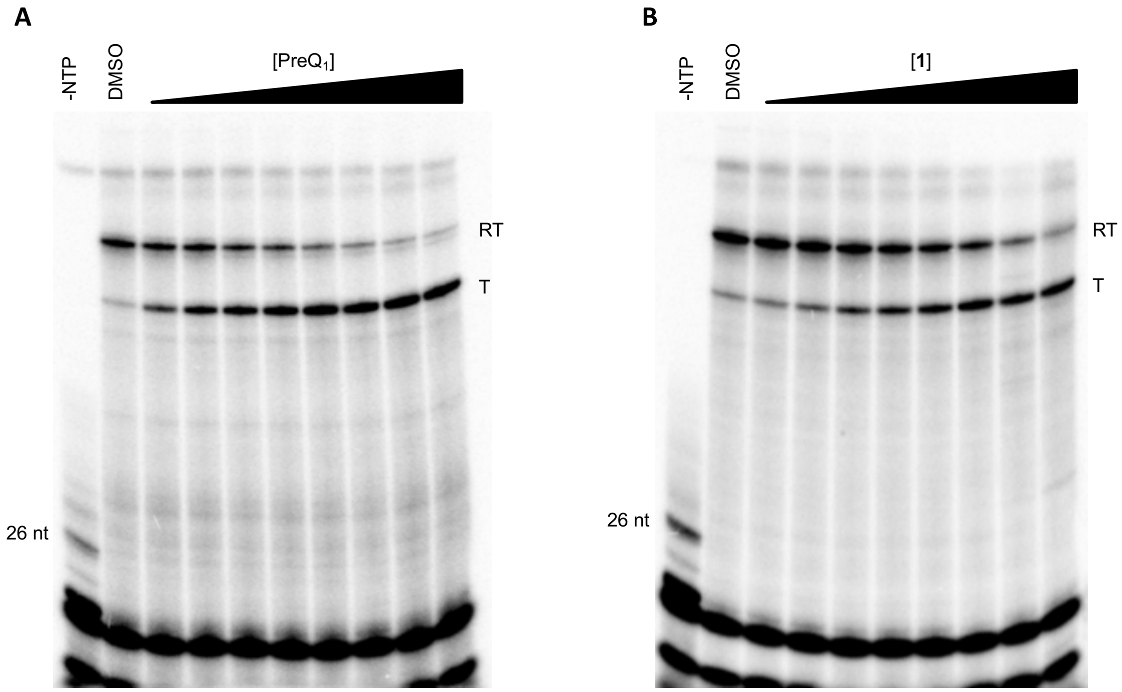

Supplementary Figure 6. Representative gel images of transcription termination assays of the *Ss* $PreQ_1$-RS template in the presence of increasing concentrations of $PreQ_1$ (**A**) and **1** (**B**) compared to a DMSO control. Bands corresponding to the read through transcription product (RT) and terminated transcription product (T) are indicated. Experiments were performed in triplicate to confirm reproducibility.

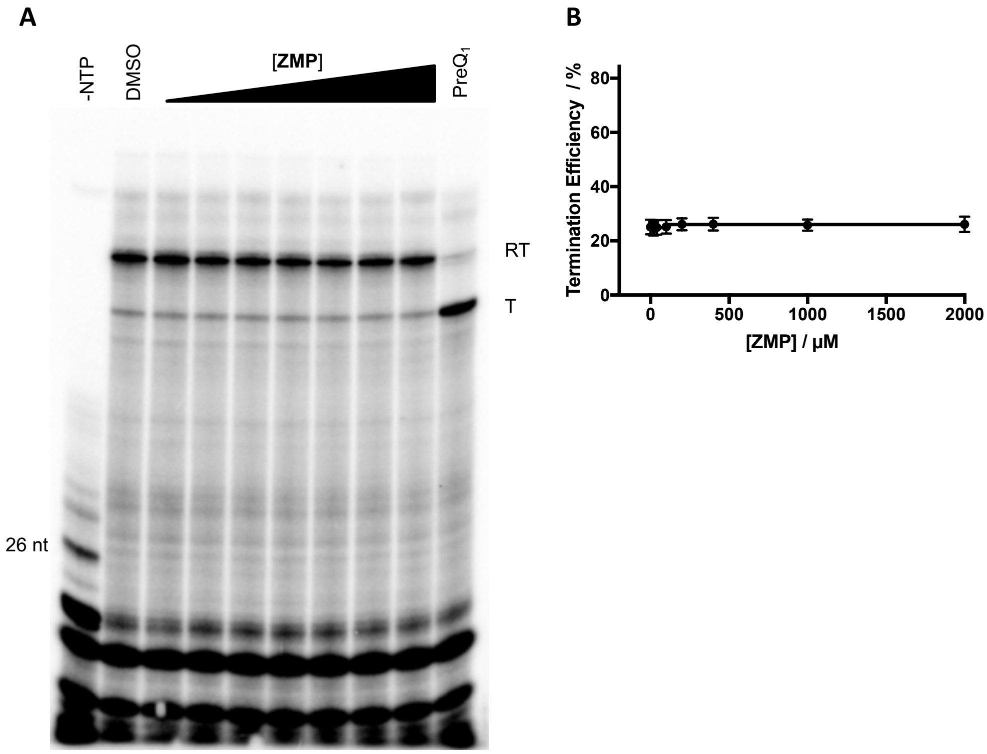

Supplementary Figure 7. (A) Representative gel images of transcription termination assays of the *Ss* $PreQ_1$-RS template in the presence of increasing concentrations of ZMP compared to DMSO (negative) and $PreQ_1$ (positive) controls. Bands corresponding to the read through transcription product (RT) and terminated transcription product (T) are indicated. **(B)** Quantification of transcription termination efficiency of increasing concentrations of ZMP based on the band intensities, where termination efficiency is determined by the background subtracted band intensity of the terminated product compared to the total band intensity of the read through and terminated products. Error bars indicate the standard deviation determined from three independent measurements.

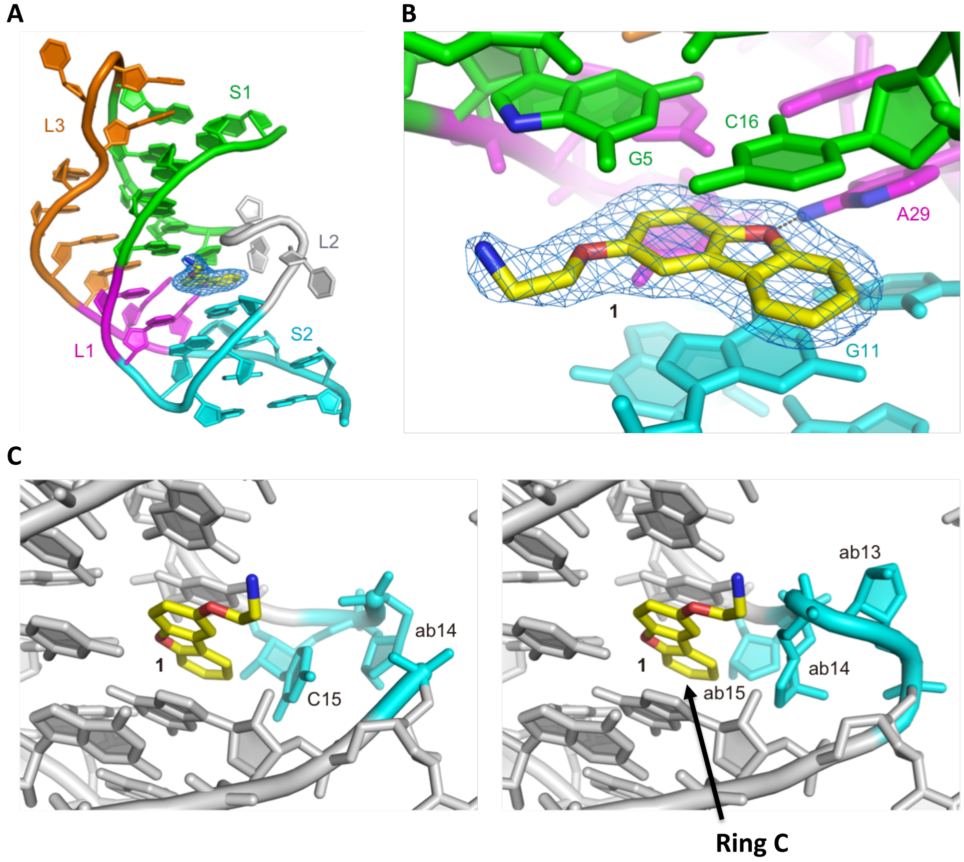

Supplementary Figure 8. The co-crystal structure of the ab13_14_15 *Tt* $PreQ_1$ riboswitch aptamer complexed with **1**. **(A)** Overall structure of the complex. Secondary structure elements are shown in the same color codes as in Figure 6. The unbiased $|F_o| - |F_c|$ electron density map for **1** is colored blue and contoured at 3.0 σ. **(B)** Close up view of the ligand binding site. The nucleotides that interact with **1** are labeled, and a hydrogen bond is shown as a dotted line. **(C)** Structural comparison of the L2 in the ab13_14-**1** and ab13_14_15-**1** structures. **1** is colored yellow, and the nucleotides at position 13, 14, and 15 are in cyan.

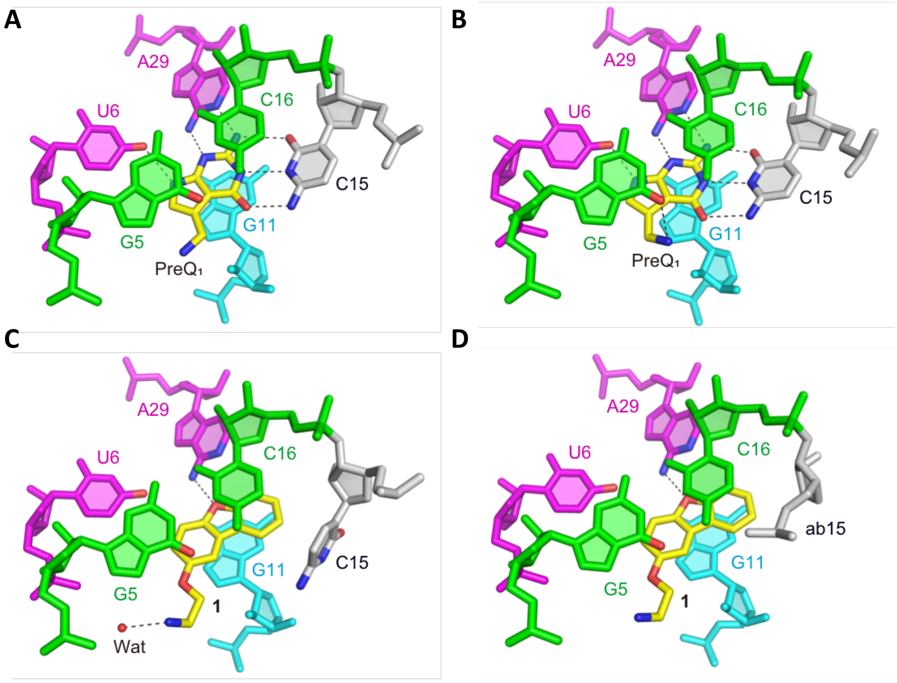

Supplementary Figure 9. Compound binding site in the *Tt* $PreQ_1$ riboswitch aptamer. **(A)** $PreQ_1$ binding site of the WT-$PreQ_1$ structure. **(B)** $PreQ_1$ binding site of the ab13_14-$PreQ_1$ structure. **(C)** **1** binding site of the ab13_14-**1** structure. **(D)** **1** binding site of the ab13_14_15-**1** structure. The ligands are colored yellow, and nucleotides contact the ligand are shown in the same color codes in Figure 6. Hydrogen bonds are indicated as dotted lines.

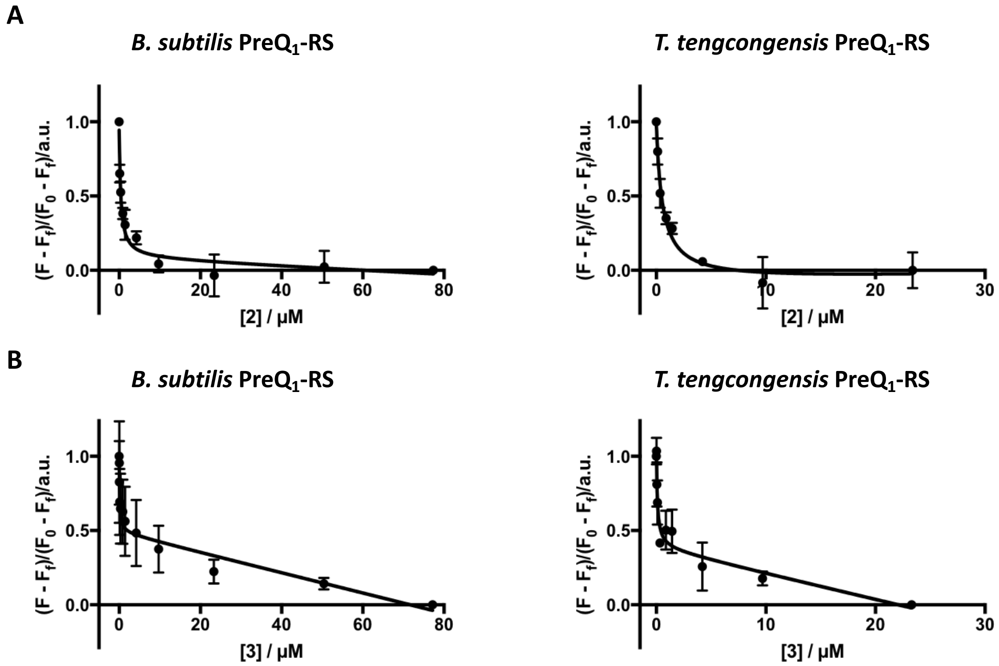

Supplementary Figure 10. Fluorescence intensity assays of 5′-Cy5-labeled *Bs*PreQ_1-RS or 5′-AlexaFluor 647-labeled *Tt*PreQ_1-RS RNA in the presence of increasing concentration of **2** (**A**) or **3** (**B**) used to determine binding affinities. Error bars indicate the standard deviation determined from three independent measurements.

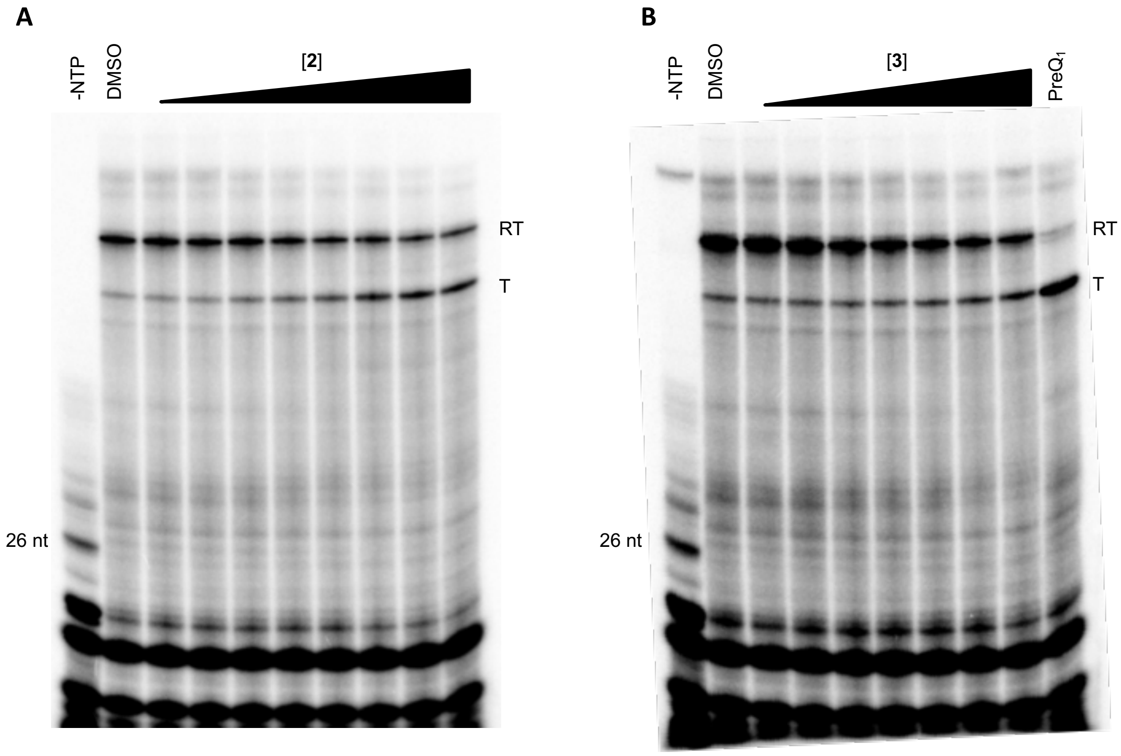

Supplementary Figure 11. Representative gel images of transcription termination assays of the *Ss* $PreQ_1$-RS template in the presence of increasing concentrations of **2** (**A**) and **3** (**B**) compared to a DMSO control. Bands corresponding to the read through transcription product (RT) and terminated transcription product (T) are indicated. Experiments were performed in triplicate to confirm reproducibility.

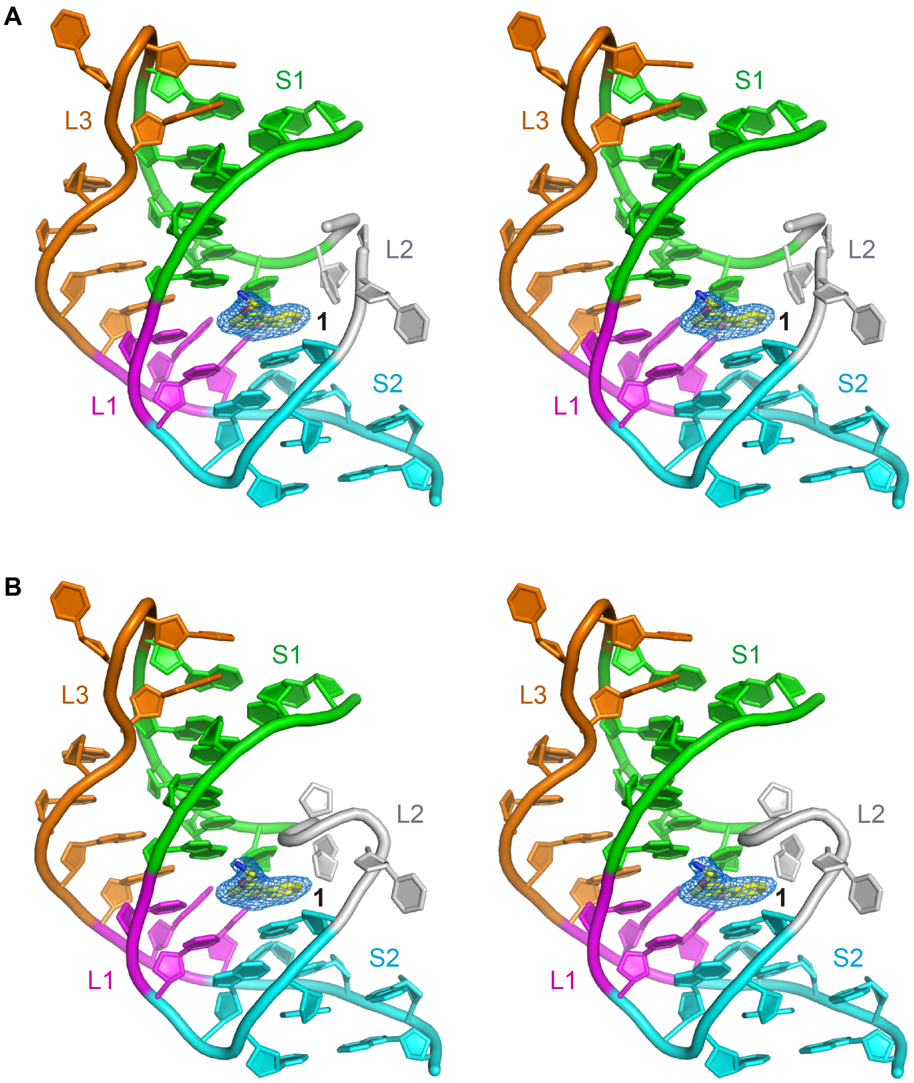

Supplementary Figure 12. Stereo images of the overall structure of ab13_14 (**A**) and ab13_14_15 (**B**) *Tt* PreQ$_1$ riboswitch aptamer complexed with **1**. S1, S2, L1, L2, and L3 are colored green, cyan, magenta, gray, and orange, respectively. The nucleotides between L3 and S2, which interact with L1, are also in magenta. Unbiased $|F_o| - |F_c|$ electron density map for the compound **1** (blue mesh) contoured at 3.0 σ.

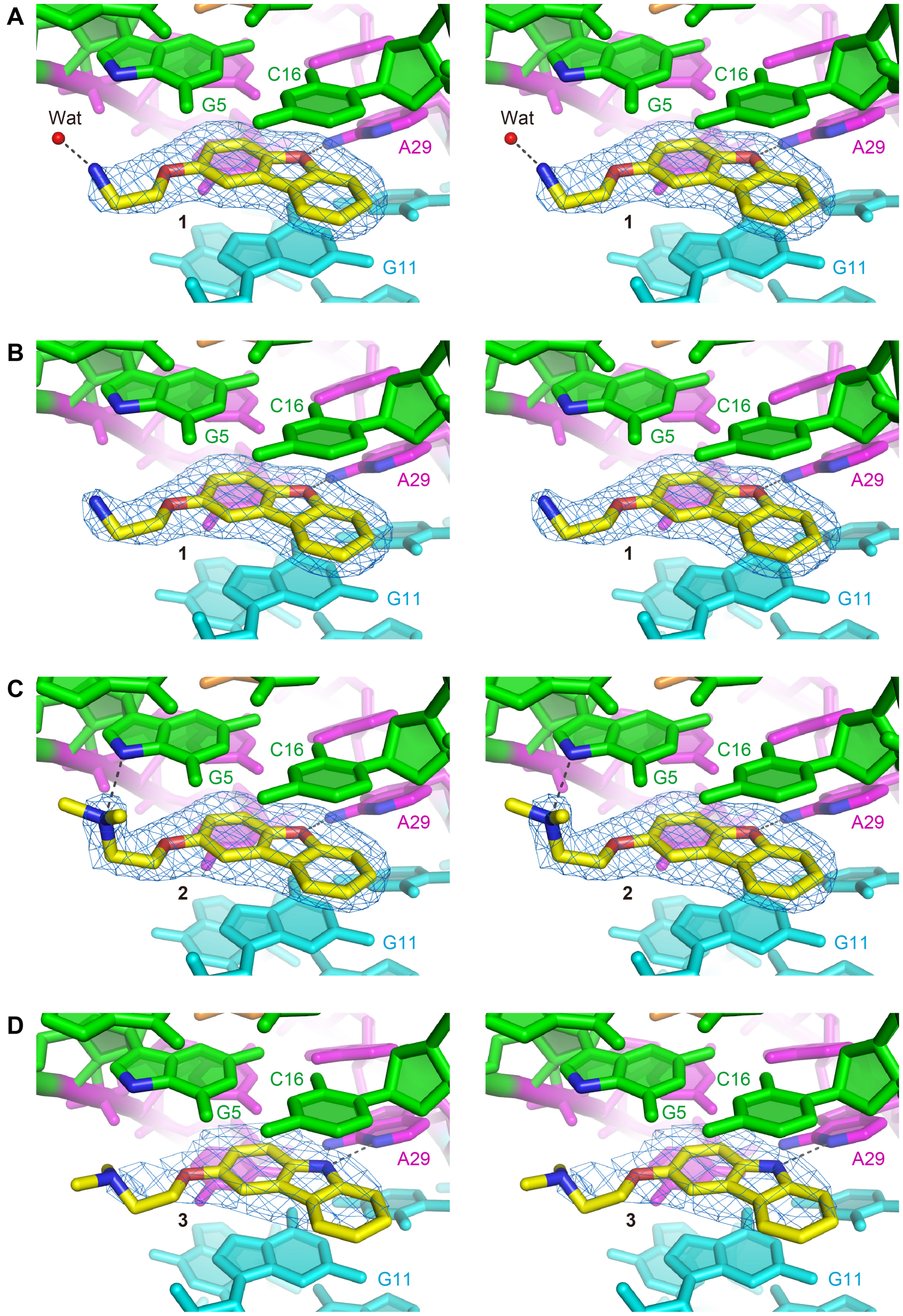

Supplementary Figure 13. Stereo images of the compound binding site of the ab13_14-**1** (**A**), ab13_14_15-**1** (**B**), ab13_14-**2** (**C**), and ab13_14_15-**3** (**D**) co-crystal structures. Unbiased $|F_o| - |F_c|$ electron density map for the compounds (blue mesh) contoured at 3.0 σ. Hydrogen bonds are indicated as dotted lines.

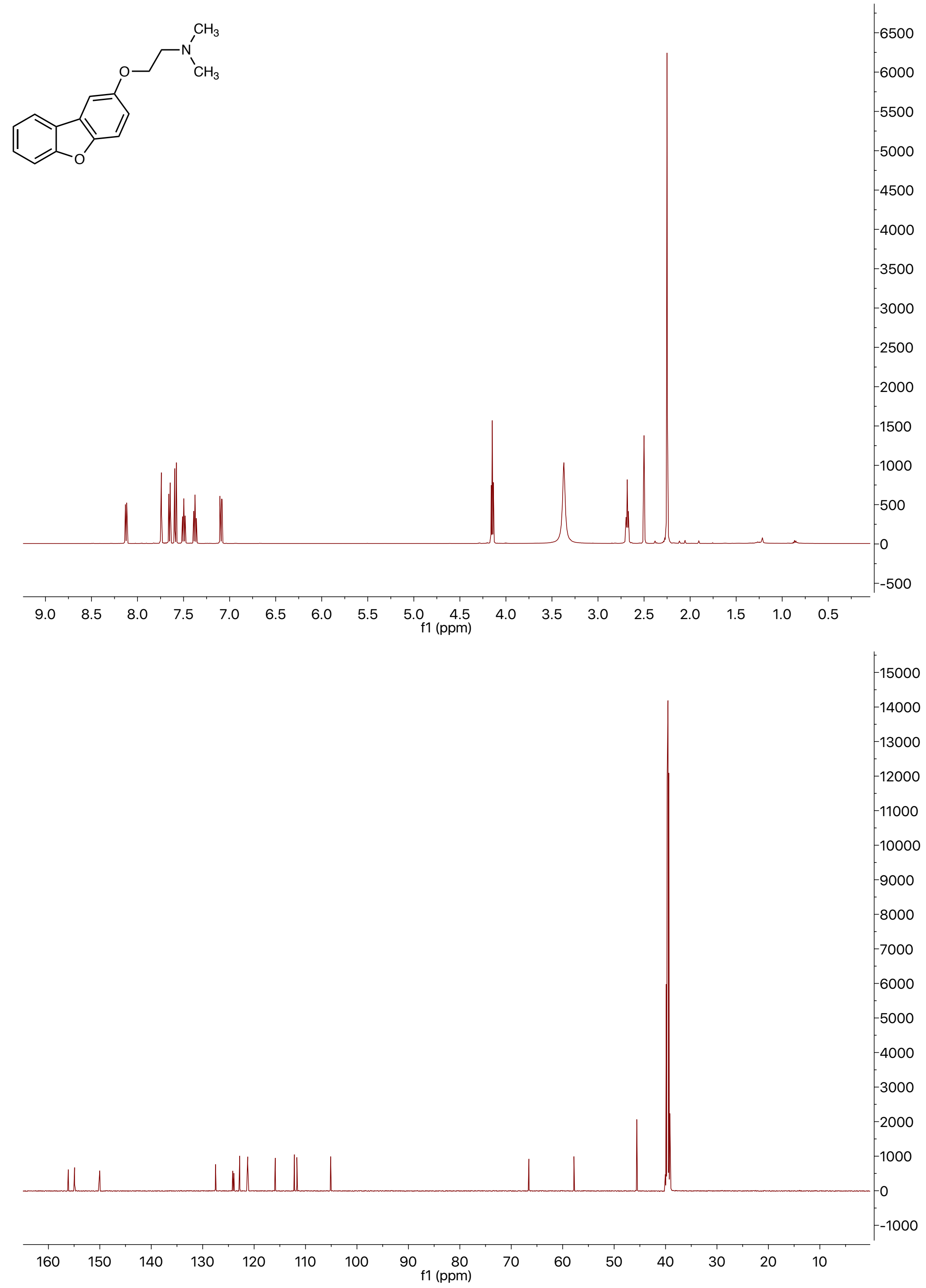

Supplementary Figure 14. ^{1}H NMR (top) and ^{13}C NMR (bottom) of dibenzofuran **2.**

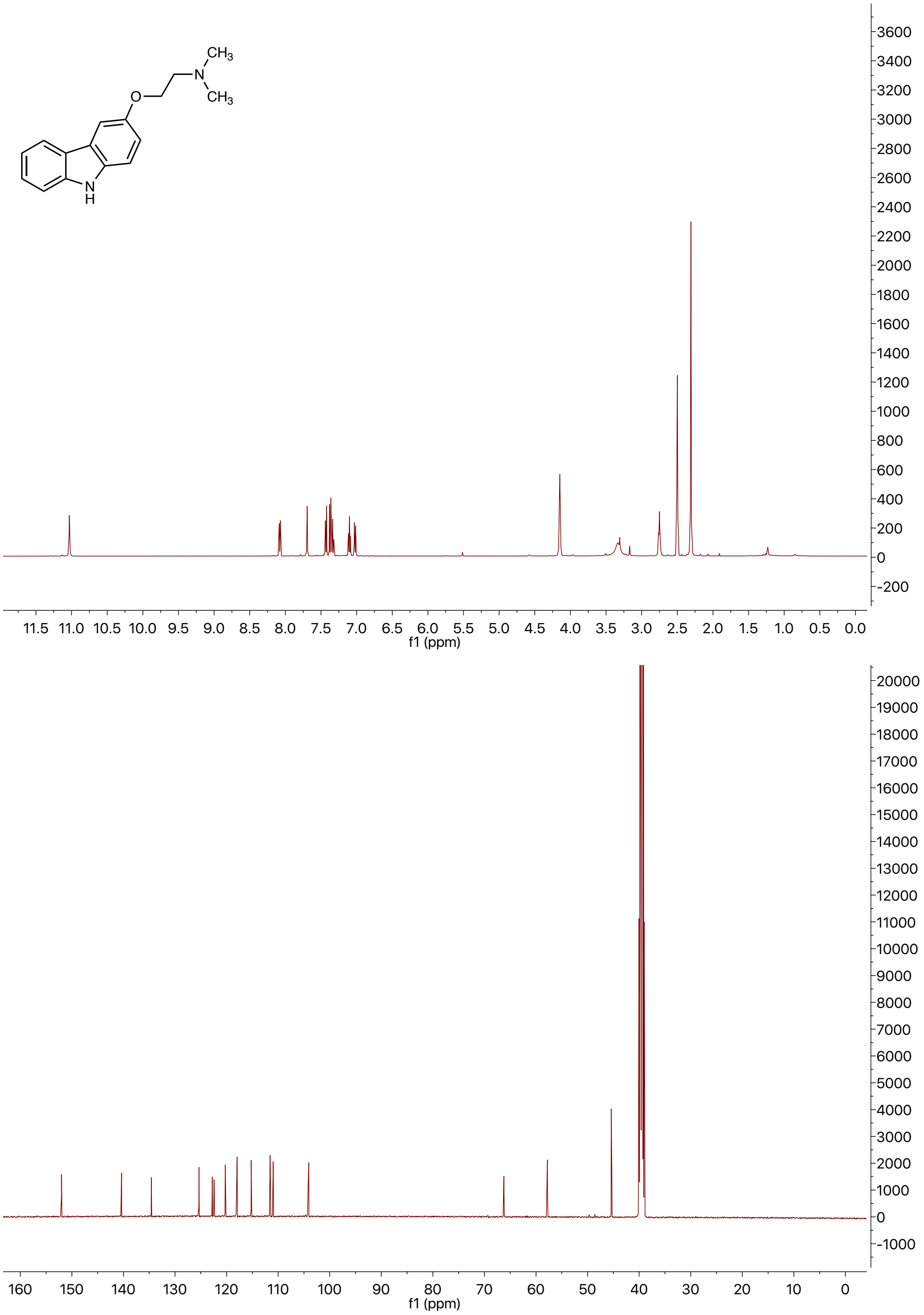

Supplementary Figure 15. ^{1}H NMR (top) and ^{13}C NMR (bottom) of carbazole **3.**

Supplementary References

1 Ku, S. K. *et al*. Vascular barrier protective effects of 3-N- or 3-O-cinnamoyl carbazole derivatives. *Bioorg Med Chem Lett* **25**, 4304-4307, doi:10.1016/j.bmcl.2015.07.079 (2015).

2 Connelly, C. M., Boer, R. E., Moon, M. H., Gareiss, P. & Schneekloth, J. S., Jr. Discovery of Inhibitors of MicroRNA-21 Processing Using Small Molecule Microarrays. *ACS Chem Biol* **12**, 435-443, doi:10.1021/acschembio.6b00945 (2017).

3 Kozomara, A. & Griffiths-Jones, S. miRBase: annotating high confidence microRNAs using deep sequencing data. *Nucleic Acids Res* **42**, D68-73, doi:10.1093/nar/gkt1181 (2014).

4 Luedtke, N. W., Liu, Q. & Tor, Y. RNA-ligand interactions: affinity and specificity of aminoglycoside dimers and acridine conjugates to the HIV-1 Rev response element. *Biochemistry-Us* **42**, 11391-11403, doi:10.1021/bi034766y (2003).

5 Brown, J. A., Valenstein, M. L., Yario, T. A., Tycowski, K. T. & Steitz, J. A. Formation of triple-helical structures by the 3'-end sequences of MALAT1 and MENbeta noncoding RNAs. *Proc Natl Acad Sci U S A* **109**, 19202-19207, doi:10.1073/pnas.1217338109 (2012).

6 Fleming, A. M., Ding, Y., Alenko, A. & Burrows, C. J. Zika Virus Genomic RNA Possesses Conserved G-Quadruplexes Characteristic of the Flaviviridae Family. *Acs Infect Dis* **2**, 674-681 (2016).

7 Kumari, S., Bugaut, A., Huppert, J. L. & Balasubramanian, S. An RNA G-quadruplex in the 5' UTR of the NRAS proto-oncogene modulates translation. *Nat Chem Biol* **3**, 218-221, doi:10.1038/nchembio864 (2007).

8 Garavis, M. *et al*. Discovery of selective ligands for telomeric RNA G-quadruplexes (TERRA) through 19F-NMR based fragment screening. *ACS Chem Biol* **9**, 1559-1566, doi:10.1021/cb500100z (2014).

9 Grohar, P. J. *et al*. Functional Genomic Screening Reveals Splicing of the EWS-FLI1 Fusion Transcript as a Vulnerability in Ewing Sarcoma. *Cell Rep* **14**, 598-610, doi:10.1016/j.celrep.2015.12.063 (2016).

10 Agarwala, P., Pandey, S. & Maiti, S. Role of G-quadruplex located at 5' end of mRNAs. *Biochimica et biophysica acta* **1840**, 3503-3510, doi:10.1016/j.bbagen.2014.08.017 (2014).

11 Calabrese, D. R. *et al*. Characterization of clinically used oral antiseptics as quadruplex-binding ligands. *Nucleic Acids Res* **46**, 2722-2732, doi:10.1093/nar/gky084 (2018).

12 Felsenstein, K. M. *et al*. Small Molecule Microarrays Enable the Identification of a Selective, Quadruplex-Binding Inhibitor of MYC Expression. *ACS Chem Biol* **11**, 139-148, doi:10.1021/acschembio.5b00577 (2016).

13 Felsenstein, K. M. *et al*. Small Molecule Microarrays Enable the Identification of a Selective, Quadruplex-Binding Inhibitor of MYC Expression. *ACS Chem Biol* **11**, 139-148 (2016).

14 Rankin, S. *et al*. Putative DNA quadruplex formation within the human c-kit oncogene. *J Am Chem Soc* **127**, 10584-10589, doi:10.1021/ja050823u (2005).
